# Hub Protein Controversy: Taking a Closer Look at Plant Stress Response Hubs

**DOI:** 10.3389/fpls.2018.00694

**Published:** 2018-06-05

**Authors:** Katy Vandereyken, Jelle Van Leene, Barbara De Coninck, Bruno P. A. Cammue

**Affiliations:** ^1^Centre of Microbial and Plant Genetics, KU Leuven, Heverlee, Belgium; ^2^VIB Center for Plant Systems Biology, Ghent, Belgium; ^3^Department of Plant Biotechnology and Bioinformatics, Ghent University, Ghent, Belgium; ^4^Division of Crop Biotechnics, KU Leuven, Heverlee, Belgium

**Keywords:** hub proteins, protein-protein interaction networks, plant interactome, plant stress response, hub protein identification

## Abstract

Plant stress responses involve numerous changes at the molecular and cellular level and are regulated by highly complex signaling pathways. Studying protein-protein interactions (PPIs) and the resulting networks is therefore becoming increasingly important in understanding these responses. Crucial in PPI networks are the so-called hubs or hub proteins, commonly defined as the most highly connected central proteins in scale-free PPI networks. However, despite their importance, a growing amount of confusion and controversy seems to exist regarding hub protein identification, characterization and classification. In order to highlight these inconsistencies and stimulate further clarification, this review critically analyses the current knowledge on hub proteins in the plant interactome field. We focus on current hub protein definitions, including the properties generally seen as hub-defining, and the challenges and approaches associated with hub protein identification. Furthermore, we give an overview of the most important large-scale plant PPI studies of the last decade that identified hub proteins, pointing out the lack of overlap between different studies. As such, it appears that although major advances are being made in the plant interactome field, defining hub proteins is still heavily dependent on the quality, origin and interpretation of the acquired PPI data. Nevertheless, many hub proteins seem to have a reported role in the plant stress response, including transcription factors, protein kinases and phosphatases, ubiquitin proteasome system related proteins, (co-)chaperones and redox signaling proteins. A significant number of identified plant stress hubs are however still functionally uncharacterized, making them interesting targets for future research. This review clearly shows the ongoing improvements in the plant interactome field but also calls attention to the need for a more comprehensive and precise identification of hub proteins, allowing a more efficient systems biology driven unraveling of complex processes, including those involved in stress responses.

## Introduction

Plants are continuously exposed to different kinds of environmental stresses. These can be abiotic, such as drought, salinity, non-optimal light or temperature conditions, or biotic in origin, such as pathogen or herbivore attacks. The plant stress response is therefore highly complex and although the molecular mechanisms that occur upon stress detection have been extensively studied, much remains to be unraveled (Bari and Jones, 2009; Dodds and Rathjen, 2010; Atkinson and Urwin, 2012; Duque et al., 2013; Rejeb et al., 2014). In the current era of systems biology driven research it is important to analyze systems as a whole, rather than their individual constituents. Consequently, studying the plant protein interactome—i.e., the set of physical protein interactions in a cell—can provide valuable new insights into biological processes (Ideker et al., 2001; Garbutt et al., 2014; Mine et al., 2014; Sheth and Thaker, 2014).

**Figure 1:**
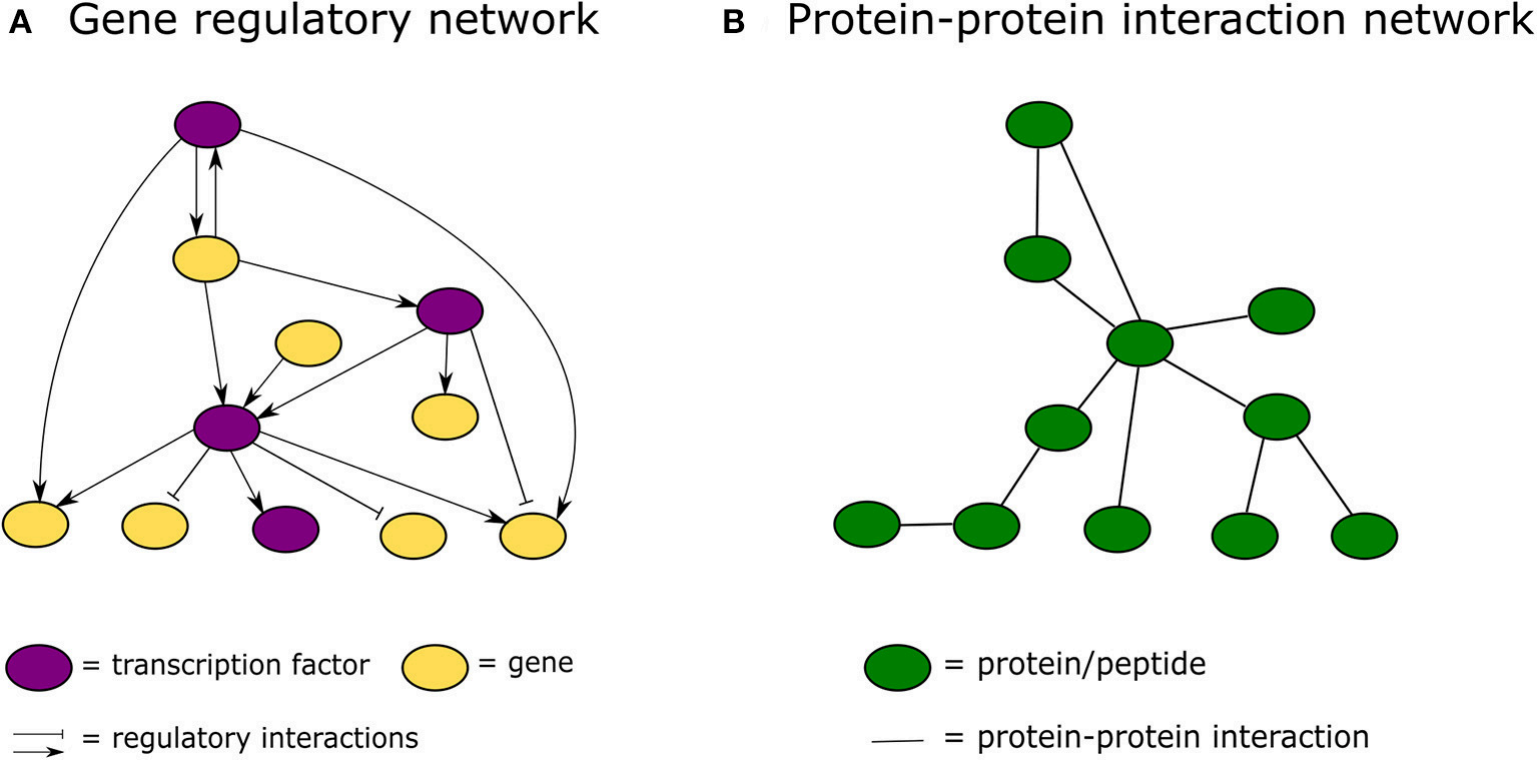
Graphical representation of a gene regulatory network vs. a protein-protein interaction network. **(A)** In a gene regulatory network nodes represent genes or proteins and lines between them regulatory interactions. **(B)** In a protein-protein interaction network nodes always represent proteins and the connecting lines physical protein-protein interactions.

**Figure 2:**
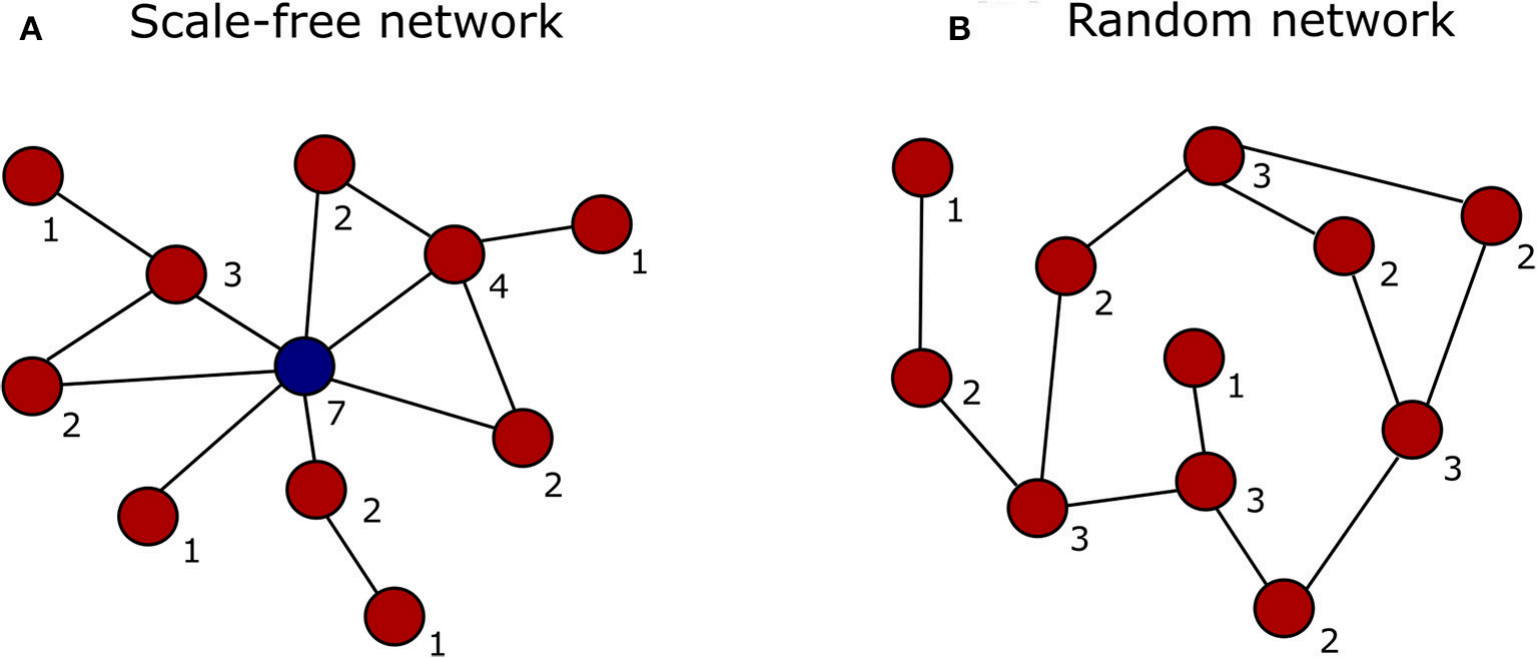
Graphical representation of a scale-free vs. a random network. The degree (number of interactions) of each node is indicated by the digit below the node. **(A)** In a scale-free network the majority of nodes interact with just a few other nodes (red), while only some interact with many other nodes and represent the network hubs (blue). **(B)** In a random network the nodes (red) are connected with a uniform probability, resulting in most nodes having the same number of connections.

In the past decade, advances in high-throughput proteomics and interactomics technologies resulted in an accumulation of increasingly accurate plant protein-protein interaction (PPI) data (Morsy et al., 2008; Braun et al., 2013; Xing et al., 2016). These data are often represented graphically in the form of PPI networks, not to be confused with genetic interactions or gene regulatory networks used to elucidate how genes function as a network in biological processes (Figure 1; Zhang et al., 2010; Krouk et al., 2013). Studies show that most biological networks including PPI networks, are scale-free, meaning that only a limited number of proteins interact with numerous others (Figure 2; Albert, 2005; Vallabhajosyula et al., 2009). Because of their high connectivity, these proteins are called hub proteins (or hubs) and they are of critical importance to PPI networks and whole biological systems (Higurashi et al., 2008). Indeed, when a hub is eliminated or interfered with, the structure of the biological networks will change drastically, having a vast impact on the organismal fitness (He and Zhang, 2006). Specifically in plant stress response-related networks, hubs are assumed to play an essential role in the signal transduction cascade following stress detection and loss-of-function mutants often show abnormal stress responses (Dietz et al., 2010). Still, despite their great importance and major advances in the interactome field, there is a lack of clarity regarding hub proteins, made evident by inconsistent definitions, characteristics, and classification systems. This review critically assesses the current knowledge in the plant interactome field, focusing specifically on plant stress response related hubs in large-scale plant PPI networks.

## Characterizing hub proteins: definitions and properties

### Current hub definitions

While the original definition of the term “hub” refers to the solid central part of a wheel into which spokes are inserted, nowadays a hub refers to any kind of system's center around which various other system components revolve or from which they radiate, and as such are interconnected. This definition was translated to molecular biology, where hub proteins were defined as highly connected central nodes in a systematic scale-free PPI network, having numerous interaction partners and connecting many network modules (Han et al., 2004; Ekman et al., 2006; Haynes et al., 2006; Tsai et al., 2009; Patil et al., 2010; Bertolazzi et al., 2013; Ota et al., 2016). Biological networks such as PPI networks often adopt a scale-free architecture, meaning that the vast majority of network nodes have a low degree of interactions while relatively few have high connectivity, the latter termed hubs (Figure 2; Jeong et al., 2001; Rangarajan et al., 2015). However, the exact number of interactions needed for a protein to be labeled as a hub, also called the degree threshold, differs between PPI studies. Some sources cite a fixed degree cutoff of 5 (Jeong et al., 2001; Han et al., 2004; Patil and Nakamura, 2006), 8 (Ekman et al., 2006), 10 (Haynes et al., 2006), 20 (Aragues et al., 2007), or 50 interactors (Mukhtar et al., 2011). Others take a floating cutoff as a top percentage of high degree nodes, with hubs most often defined as the top 10% of proteins with the highest number of interactors (Batada et al., 2006; Dosztányi et al., 2006; Jin et al., 2007). Hence there seems to be no consensus on the degree threshold. At present, the term “hub” is increasingly used more loosely, and even often incorrectly, as it has become a fashionable label for any central signaling network protein of some importance, independent from the fact that the concerned protein may or may not participate in numerous protein interactions. This unavoidably leads to an increase of per definition incorrectly labeled hubs and a growing confusion.

For the transition between high-degree nodes and intermediate or low degree nodes in general scale-free PPI networks a threshold of more than 5 interactions has previously been stated (Vallabhajosyula et al., 2009). However, this seems very low for hubs defined as the most highly connected proteins in comprehensive PPI networks. Indeed when looking at large-scale plant PPI networks, many proteins have more than 5 interactors, indicating that this is not a justified threshold. Some studies also suggest to differentiate between small (6–10), intermediate (11–50), major (51–100), and super hubs (>100 interactions). However, as PPI networks become more extensive, more interactions will be found for a single protein, making any fixed cutoff seemingly less suitable. In the view that hub function is a property of the given interactome network and not something that can be defined at the level of individual proteins, a floating cutoff can seem more relevant and flexible (Dosztányi et al., 2006). Still, this last method can also be subjective as to what exact top percentage should be chosen and the minimum degree for hubs will also depend on the size and connectivity of the network of interest. The quality and origin of the PPI data presents additional challenges for hub protein identification based on interaction degree (Andorf et al., 2013; Bertolazzi et al., 2013). A general cutoff for plant hub proteins is therefore not straightforward.

### Network properties

Besides their **high connectivity**, hub proteins are also often described as being characterized by other network properties (Table 1), with centrality being the most essential and referring to their central position in relationship to other proteins in the network (McCormack et al., 2015). There are many different measures for **centrality** in biological networks (De Arruda et al., 2014; Vella et al., 2017), but the most commonly used measures are (i) “degree centrality”, simply defined by the number of interactions a protein has in a network, (ii) “betweenness centrality”, based on the shortest paths between network proteins and referring to a protein's frequency in mediating connections to other proteins, and (iii) “eigenvector centrality”, referring to the influence of the protein in a network not strictly through the number of connections but through the importance of these connections (Liseron-Monfils and Ware, 2015; McCormack et al., 2015).

**Table 1:** Network and structural properties often seen as hub-defining.

**NETWORK PROPERTIES**	
**High connectivity**	**Hubs physically interact with numerous other proteins, but there is no minimum degree consensus, fixed or floating cutoffs are used**
**Centrality**	**Several centrality measures reflect a hub protein's increased ability and importance in mediating connections between proteins**
**Pleiotropy**	**Hubs often participate in several cellular processes and a number of phenotypes can be observed as a consequence of gene knockout**
**Interconnectivity**	**An increasing amount of studies define hubs as highly interconnected proteins**
**Co-expression bimodality**	**Hubs can be divided into two classes, either showing low co-expression (date hubs) of high co-expression (party hubs) with their interactors**
**STRUCTURAL PROPERTIES**	
**Length**	**Hubs are often long proteins with multiple repeated or binding domains, although short hub proteins also exist**
**Ordered binding domains**	**Many large hub proteins have multiple binding domains, corresponding with ordered or globular regions of the proteins**
**Intrinsic disorder regions**	**The presence of disordered regions lacking a unique structure and allowing protein flexibility is seen as a common feature of hub proteins**
**Highly charged surfaces**	**Small hub proteins often have highly charged surfaces to facilitate binding with multiple partners**
**Single/multiple interfaces**	**Hubs can be divided into two classes, depending on the presence of a single (single interface hubs) or multiple interaction sites (multiple interface hubs)**

Hub proteins with a high degree of connectivity and centrality in a PPI network are also often said to be essential for PPI network topology and interactome functionality and stability (Jeong et al., 2001; Vallabhajosyula et al., 2009; Dietz et al., 2010). This also implies that hub proteins can be weak points in PPI networks, with the potential of disrupting an entire system when, for example, deleted or targeted by pathogen attacks (Mukhtar et al., 2011; Garbutt et al., 2014; Weβling et al., 2014; Ota et al., 2016). Still, the relationship between hub degree, centrality and essentiality has been heavily debated. At first, Jeong et al. (2001) concluded that highly connected proteins with a central role in networks are more likely to be encoded by essential genes, that when deleted cause lethal phenotypes, leading to the so-called lethality-centrality rule. However, more recently this conclusion was reported to be incorrect and a consequence of using heavily biased datasets available at the time of the original observation (Yu et al., 2008). When less systematically biased datasets are used, the correlation between centrality and lethality is replaced by one between centrality and **pleiotropy**. The number of physical interactions mediated by a protein thus seems to be better correlated with the number of cellular processes in which it participates than with its essentiality/lethality (Yu et al., 2008). Several studies indeed indicated that hubs often participate in essential complexes and processes and mediate essential interactions, therefore affecting many phenotypic traits when knocked-out, but not necessarily resulting in lethality (He and Zhang, 2006; Zotenko et al., 2008; Song and Singh, 2013).

Another property contributed to hub proteins in some studies is low **interconnectivity** as compared to non-hub proteins (Maslov and Sneppen, 2002; Vallabhajosyula et al., 2009). However paradoxically, an increasing amount of studies define PPI network hubs as highly (inter)connected proteins, indicating that hubs are often together in larger complexes (de Folter, 2005; Popescu et al., 2007; Hunt et al., 2008; Causier et al., 2012; Lumba et al., 2014; Piya et al., 2014; McCormack et al., 2015).

Lastly, co-expression between hub proteins and their interaction partners is often found to have a statistically significant bimodal distribution, termed **co-expression bimodality** (Vallabhajosyula et al., 2009). This implies the existence of two classes of hub proteins, one with low and another with high averaged interactor co-expression values, referred to as date and party hubs, respectively (Han et al., 2004). Party (or static) hubs are usually larger proteins that are part of functional protein complexes and interact with multiple partners simultaneously and continuously, assuming co-expression. Date (or dynamic) hubs on the other hand are usually smaller proteins that interact more transiently with one or two of their interaction partners at different time points and locations, therefore showing low co-expression. Party hubs generally act as central parts of functional protein complexes, while date hubs function as bridges between network modules (Han et al., 2004; Yu et al., 2008). However, the date/party hub classification is also quite controversial and argued not to be comprehensive enough (Batada et al., 2006, 2007; Agarwal et al., 2010; Chang et al., 2013).

### Structural properties

Concerning structural properties, it is somewhat unclear what is needed for a protein to evolve into a hub, making it able to recognize and bind many different partners. However, some structure-related properties are statistically more often ascribed to and thus suggested to be characteristic for hub proteins (Table 1). Evidently, the proportion of **multi-domain proteins or long proteins** with repeating domains, by which they can effectively bind different partners, is significantly larger amongst proteins labeled as hubs than in non-hubs. These protein binding domains are usually associated with **ordered or globular regions** of the proteins (Ekman et al., 2006). Conversely, several studies suggest that the presence of disordered regions is a common feature of eukaryotic hub proteins (Haynes et al., 2006; Vincent and Schnell, 2016). **Intrinsically disordered** proteins (IDPs) or protein regions (IDPRs) are (segments of) proteins that lack a unique 3D structure, they do not completely fold and remain flexible (Kim et al., 2008). IDPRs facilitate multiple protein recognition and binding by acting as adaptable extended interaction surfaces. Short linear motifs (SLiMs) are often central to IDPRs. These short stretches of protein sequence, typically 3–11 residues long, mediate interactions through inducing secondary structure formation upon binding with a structured partner or globular protein domain and are especially relevant in signaling networks with high connectivity proteins (O'Shea et al., 2017). Hence IDPRs allow for high specificity, obtained through adopting an ordered conformation upon partner binding, coupled with low affinity (Uversky, 2013, 2016; Sun et al., 2014). These regions usually evolve through the expansion of internal repeat regions and exhibit low amino acid complexity (Dosztányi et al., 2006). Some smaller hub proteins, however, are too short to have multiple binding domains and have very few or no disordered regions. Many of these small hubs instead have **highly charged surfaces**, including arginine, tyrosine, histidine, and methionine, facilitating binding with multiple partners (Patil and Nakamura, 2006, 2007; Patil et al., 2010).

These structural hub characteristics gave rise to a second commonly used classification for hub proteins, differentiating between **single interface hubs** (SIHs) and **multiple interface hubs** (MIHs), depending on the presence of a single or multiple interaction sites, respectively, to bind their partners. There is a strong but not complete overlap between the SIH/MIH and date/party classification of hub proteins, but even combined they are argued not the be comprehensive enough as general hub classification systems (Andorf et al., 2013).

## Identifying hubs by unravelling the plant interactome: overcoming challenges through the use of complementary approaches

### Challenges in the interactome field

During evolution, higher organisms have developed immensely complex mechanisms and signaling pathways that allow them to respond to a wide variety of changing environmental conditions, pathogen attacks, and developmental challenges (Verma et al., 2016). In order to respond optimally to specific challenges whilst wasting a minimum of energy, these systems are also tightly regulated (Tomé et al., 2014). Partly due to this inherent complexity, many genes and proteins still remain unknown, uncharacterized or the understanding of their function is incomplete. Using interactomics-based approaches in research could help fill in these gaps, not in the least in further unraveling plant stress responses. Plants harbor vast and dynamic protein networks, with a number of protein interaction pairs estimated to range from 75,000 to 299,000 in proteomes of 30,000–40,000 proteins (Arabidopsis Interactome Mapping Consortium, 2011; Sheth and Thaker, 2014). Moreover, all these proteins have diverse physicochemical properties, expression levels, subcellular localizations and reaction kinetics. As a result, no single PPI identification approach can at present build a complete map of the plant interactome (McCormack et al., 2015). Additionally, interactome studies in plants are somewhat lagging behind the leading studies done in yeast, animal and human models (Rolland et al., 2014; Van Leene et al., 2015; Mehta and Trinkle-Mulcahy, 2016). Large-scale plant PPI networks have mostly been reconstructed during the past decade, but the data are still far from depicting global PPI maps in a plant cell (Hao et al., 2016). Plant PPI maps will thus become increasingly accurate, reliable, and extensive in the upcoming years, thereby also allowing easier and more precise identification of hub proteins. To this end, several complementary approaches, including a range of computational prediction methods and various experimental wet lab techniques, are being used and developed.

### Computational approaches

*In silico* predictions usually yield large PPI networks that are often based on evolutionary conservation (e.g., sequence alignment), protein structural information (e.g., physicochemical properties, primary structures, 3D structures, docking ability, protein domain interactions), co-expression of the corresponding genes and/or integration of various available datasets (Cui et al., 2008; De Bodt et al., 2012; Braun et al., 2013; Zahiri et al., 2013; Vella et al., 2017). Many computationally predicted interactome maps are based on known sequence similarities between interacting proteins in other model species, called conserved interacting orthologs or interologs (Yu et al., 2004; Windram et al., 2014). Interolog mapping was used to build the first truly large-scale plant interactome map for *Arabidopsis thaliana* (Geisler-Lee et al., 2007) and later also for several other plant and crop species (Tables 2, 3). Genetic algorithms, mostly domain-based like the ENTS algorithm (Elucidating Network Topology with Sequence), are also used for PPI network reconstruction. This approach uses pairwise combination of conserved domains and predictions of subcellular protein localization as input features and have already predicted large PPI networks for several organisms, including *A. thaliana* and *Populus trichocarpa* (Rodgers-Melnick et al., 2013; Hao et al., 2016).

**Table 2:** Historical overview of large-scale plant interactome studies.

**Year**	Technique^*^	**Species**	**Research topic**	**Proteins in network^#^**	**Interactions in network**	**Hubs mentioned°**	**References**
2006	Protein purification	*O. sativa*	Virus-host protein complexes	224	ND	No	Brizard et al., 2006
2006	Y2H + AP-MS	*H. vulgare*	14-3-3 interactome	155	~500	No	Schoonheim et al., 2006
2007	Computational	*A. thaliana*	Arabidopsis interactome	3,482	19,979	Yes	Geisler-Lee et al., 2007
2007	Protein array	*A. thaliana*	Calmodulin-related proteins	180	716	Yes	Popescu et al., 2007
2007	AP-MS	*A. thaliana*	Ubiquitinated Arabidopsis proteome	294	294	No	Maor et al., 2007
2008	Computational	*A. thaliana*	Arabidopsis interactome database	12,506	28,062	No	Cui et al., 2008
2009	Computational	*A. thaliana*	Arabidopsis interactome	1,722	3,035	No	De Bodt et al., 2009
2009	TAP-MS	*A. thaliana*	14-3-3 proteins	131	130	No	Chang et al., 2009
2009	Protein array	*A. thaliana*	MAPK target networks	580	1,280	No	Popescu et al., 2009
2009	Y2H	*O. sativa*	Protein kinase interactions	370	378	No	Ding et al., 2009
2010	sUbq	*A. thaliana*	Membrane protein interactions	179	343	No	Lalonde et al., 2010
2010	TAP-MS	*A. thaliana*	Cell cycle proteins	393	857	No	Van Leene et al., 2010
2011	Y2H	*A. thaliana*	Arabidopsis interactome	2,661	5,664	Yes	Arabidopsis Interactome Mapping Consortium, 2011
2011	Computational	*C. canephora*	Coffee interactome	939	4,587	Yes	Geisler and Fitzek, 2011
2011	Computational	*O. sativa*	Rice interactome	5,049	76,585	Yes	Gu et al., 2011
2011	Y2H	*A. thaliana*	G-protein interactome	434	1,058	No	Klopffleisch et al., 2011
2011	Computational	*A. thaliana*	Arabidopsis interactome	ND	149,900	No	Lin et al., 2011
2011	Y2H	*A. thaliana*	Plant immune system network	926	1,358	Yes	Mukhtar et al., 2011
2011	Y2H + BiFC	*O. sativa*	Rice stress response	100	77	Yes	Seo et al., 2011
2011	AP-MS	*A. thaliana*	14-3-3 proteins	106	129	No	Swatek et al., 2011
2012	Y2H	*A. thaliana*	TOPLESS	ND	655	Yes	Causier et al., 2012
2012	Computational	*O. sativa*	Rice interactome	4,567	37,112	Yes	Ho et al., 2012
2012	Computational	*B. rapa*	*B. rapa* interactome	ND	723,310	Yes	Yang et al., 2012
2013	Genetic algorithm	*A. thaliana*	Whole genome PPI network	15,964	346,020	No	Rodgers-Melnick et al., 2013
2013	Genetic algorithm	*P. trichocarpa*	Whole genome PPI network	19,321	481,253	No	Rodgers-Melnick et al., 2013
2014	AP-MS	*A. thaliana*	Qa-SNARE, membrane transport	ND	518	No	Fujiwara et al., 2014
2014	sUbq	*A. thaliana*	Membrane interactome	6.4^*^10^6^	12,102	Yes	Jones et al., 2014
2014	Y2H	*A. thaliana*	ABA signaling	138	>500	Yes	Lumba et al., 2014
2014	Y2H+BiFC	*A. thaliana*	Auxin signaling network	433	49	No	Vernoux et al., 2011
2014	Computational	*A. thaliana*	Arabidopsis-Pseudomonas interactome	>12,000	>800,000	No	Sahu et al., 2014
2014	Y2H	*A. thaliana*	Arabidopsis-pathogen	301	583	No	Weβling et al., 2014
2015	Computational	*Z. mays*	Maize interactome	6,004	49,026	Yes	Musungu et al., 2015
2015	Computational	*P. patens*	*P. patens* interactome	5,695	67,740	Yes	Schuette et al., 2015
2016	Computational	*Z. mays*	Maize interactome database	14,000	2,762,560	No	Zhu et al., 2016
2016	Computational	*M. uniflorum*	Drought responsive proteins	1,812	6,804	Yes	Bhardwaj et al., 2016
2016	Computational + Y2H + BiFC	*S. lycopersicum*	Tomato Interactome	10,626	35,7946	Yes	Yue et al., 2016
2016	Protein array	*A. thaliana*	Transcription factor interactome	2,238	3,580	No	Yazaki et al., 2016
2016	Computational	*A. thaliana*	ABA signaling	12,574	316,747	No	Zhang et al., 2016
2017	Computational	*O. sativa*	Genome-wide rice PPI network	16,895	708,819	No	Liu et al., 2017
2017	CrY2H-seq	*A. thaliana*	Transcription factor interactions	1,453	8,577	Yes	Trigg et al., 2017

* *Y2H, Yeast Two-Hybrid; (T)AP-MS, (Tandem) Affinity Purification-Mass Spectrometry; BiFC, Bimolecular Fluorescence Complementation; sUbq, split-ubiquitin; ND, not determined*.

# *Overview limited to large-scale studies resulting in networks with at least 100 proteins*.

° *Interactome studies mentioning hubs are marked with a gray background*.

**Table 3:** Details of large-scale interolog-based computational plant interactome studies defining hub proteins.

**Year**	**Species**	**Reference species**	**Proteins in network**	**Interactions in network**	**Average interactors**	**Major hub class (degree)**	**Top hub in network (degree)**	**Top hub annotation**	**References**
2007	*A. thaliana*	4	3,482	19,979	11	Medium (51–100)	At4g26840 (172)	Small ubiquitin-like modifier	Geisler-Lee et al., 2007
2011	*C. canephora*	10	939	4,587	ND	Small (3–10)	CGN-U121410 (182)	Ubiquitin family protein	Geisler and Fitzek, 2011
2011	*O. sativa*	6	5,049	76,585	29	Small (6–20)	ND (795)	ND	Gu et al., 2011
2012	*O. sativa*	11	4,567	37,112	14–15	Medium (11–50)	Os08g39140 (686)	Heat shock protein	Ho et al., 2012
2013	*B. rapa*	1	ND	740,565	71	Small (<10), some >700	Bra014387 (17,74)	Ribosomal protein	Yang et al., 2013
2015	*Z. mays*	13	6,004	49,026	16	Intermediate (10–100)	GRMZM2G118637 (797)	Ubiquitin family protein	Musungu et al., 2015
2015	*P. patens*	14	5,695	67,740	ND	Major (51–100)	ND	ND	Schuette et al., 2015
2016	*M. uniflorum*	5	1,812	6,804	7.5	ND	ND (>100)	ND	Bhardwaj et al., 2016
2016	*S. lycopersicum*	6	10,626	357,946	35	Small (10–100)	Solyc09g010630 (3,751)	Heat shock protein	Yue et al., 2016

*ND, not determined*.

Though these predicted PPIs are in many cases a necessary starting point in mapping large-scale PPI networks and provide very useful data, the approaches are not able to predict interactions between proteins without conserved domains or clear interacting domains. Also, computationally predicted networks that are based on literature data, like the interolog-based networks, are limited by what is known at a given time point and/or which data are included in the analyses and are therefore not really able to give a correct representation of the molecular connectivity. Hence, computational predictions are often seen as preliminary data that need to be verified experimentally and preferably by multiple complementary approaches to further increase their reliability. Predicted PPIs can thus give information on the interaction potential of proteins, but whether these interactions actually take place within a cell or tissue or at a given time can only be validated through experimental approaches, preferably *in planta*.

### Experimental approaches

The most common techniques for the production of experimental plant PPI data are high-throughput methods like *in vivo* yeast two-hybrid (Y2H) and split-ubiquitin (sUbq) systems, the latter specifically used for identifying membrane protein interactions (Brückner et al., 2009; Zhang et al., 2010). The first experimental so-called “proteome-scale” interactome map for *A. thaliana* (Arabidopsis Interactome I, AI-1) was composed using a systematic Y2H-based approach (Arabidopsis Interactome Mapping Consortium, 2011; Tables 2, 4). Improvements to these techniques are always ongoing, further increasing scalability and sensitivity while reducing costs (Ecker et al., 2016; Xing et al., 2016; Yachie et al., 2016). Recently, the CrY2H-seq method, a massively multiplexed Y2H method combining a Cre recombinase interaction reporter and next-generation DNA sequencing, allowing deep-coverage interactome mapping, has been presented. Using this technique to investigate *A. thaliana* transcription factor (TF) interactions, a deep-coverage *A. thaliana* TF interaction network was created (AtTFIN-1), greatly expanding the number of known plant TF interactions (Trigg et al., 2017). Other systematic approaches include classic *in vitro* protein arrays (Feilner et al., 2005; Popescu et al., 2007, 2009) or the nucleic acid programmable protein arrays (NAPPA), replacing immobilized purified proteins on the array surface by plasmid DNA and using cell-free expression systems to generate proteins (Miersch and LaBaer, 2011). The recent development of a new HaloTag-NAPPA method greatly increased protein array technology capacity, allowing proteome scale-screenings. These high-density protein arrays were also used to create an *A. thaliana* TF interactome network (Yazaki et al., 2016). Although all these experimental approaches usually give high quality results, they are based on yeast or *in vitro* systems and still require *in planta* confirmation of the interactions. Therefore, a range of targeted PPI techniques can be used for validation purposes, including fluorescence resonance energy transfer (FRET) assays (Bhat et al., 2006; Long et al., 2017), split-luciferase systems (SLS) (Fujikawa and Kato, 2007; Li et al., 2011), bimolecular fluorescence complementation (BiFC) (Miller et al., 2015), and co-immunoprecipitation (Co-IP) (Roux et al., 2011).

**Table 4:** Details of large-scale experimental plant interactome studies defining hub proteins.

**Year**	**Species**	**Technique**	**Research topic**	**Proteins in network^#^**	**Interactions in network**	**Hubs identified in network**	**References**
2007	*A. thaliana*	Protein array	Calmodulin-related proteins	180	716	Four hubs (highly interconnected clusters of proteins) containing different CaMs/CMLs	Popescu et al., 2007
2011	*A. thaliana*	Y2H	Arabidopsis-Pathogen interactome	926	1,358	Fourteen proteins with degrees higher than 50 (hubs_50_)	Arabidopsis Interactome Mapping Consortium, 2011; Mukhtar et al., 2011
2011	*O. sativa*	Y2H + BiFC	Rice stress response	100	77	Proteins with above average degree of interaction, including XA21, SUB1A, SUB1C, XB15, XB3, OsWRKY62, and XB24	Seo et al., 2011
2012	*A. thaliana*	Y2H	TOPLESS	ND	655	TPL/TPL-related (TPR) co-repressor hub	Causier et al., 2012
2014	*A. thaliana*	sUbq + sGFP	Membrane interactome	6.4*10^∧^6	12,102	Forty-six proteins with degrees higher than 70	Jones et al., 2014
2014	*A. thaliana*	Y2H	ABA signaling	138	>500	The kinases SNRK3.15/SNRK3.22 and MAP3Kδ4, the PP2C HAI1, and the bHLH TF protein AIB1	Lumba et al., 2014
2017	*A. thaliana*	CrY2H-seq	Transcription factor interactions	1,453	8,577	TCP family transcription factors	Trigg et al., 2017

*ND, not determined*.

# *Overview limited to large-scale studies resulting in networks with at least 100 proteins*.

Lastly, approaches specifically used for the identification of protein complexes in plants, include classic immunoprecipitation assays and tag-based affinity purification (AP) methods combined with mass spectrometry (MS) (Dedecker et al., 2015). Especially tandem affinity purification followed by mass spectrometry (TAP-MS) is a very powerful and often used method because of its high specificity. With this technique protein complexes have already been identified in *A. thaliana* and *O. sativa*, both in stably transformed cell suspension cultures and seedlings, and in *Medicago truncatula* hairy roots (Goossens et al., 2016). Combining TAP with quantitative MS also allowed mapping of dynamic PPI networks over the different growth zones of maize leaves (Nelissen et al., 2015). To filter out non-specific contaminants, a non-specific background interactions “black list” is often defined (Van Leene et al., 2015). However, nowadays quantitative AP-MS (q-AP-MS) methods are increasingly being used, comparing the quantity of proteins that co-purify with the bait to a negative control, in order to greatly improve confidence in identified interaction partners without losing possibly important interactors (Meyer and Selbach, 2015).

### Validation, tools, and databases

The above-mentioned techniques are all characterized by their own inherent advantages and drawbacks, regarding throughput, background, false positive and negative rates, fusion tags, biochemistry, technical demand, costs, etc. As a result, it is important to realize that different techniques can lead to the detection of different subsets of interaction partners which are not always overlapping between the different approaches. This complicates the validation of interactions via multiple approaches (Van Leene et al., 2010, 2011; Gadeyne et al., 2014; Yazaki et al., 2016). For example, though Y2H and TAP-MS have been shown to be highly complementary, it is often challenging to validate interactions identified with one method, using the other (Yu et al., 2008; Brückner et al., 2009). Extensive knowledge on the properties and issues inherent to each method and the use of proper controls during experiments is therefore essential as the differences between methods, based on the used technology (i.e., tags, reporters, read out), the type of interactions identified (i.e., stable or transient) and the interaction environment (i.e., *in vitro* or *in vivo*, in yeast or *in planta*), can all cause diverse outcomes for the interaction analyses. For detailed information on the various plant PPI identification and validation techniques, we refer to some other excellent reviews (Morsy et al., 2008; Braun et al., 2013; McCormack et al., 2015).

Nevertheless, when a PPI can be identified through multiple high-quality approaches, its reliability increases significantly and this is an important requirement for developing networks that optimally reflect the biological reality. Therefore, increasing emphasis is also being put on creating better PPI networks through complementing them with data from various other sources, including co-expression, co-localization, co-evolution, and functional data (De Bodt et al., 2009; Zahiri et al., 2013; Vella et al., 2017). Several publically available databases, resources and tools exist to retrieve, analyze and visualize plant PPI and other related data (Table 5). Since PPI reliability is crucial in building PPI networks and maps, many of the databases also provide further details and trust values to their presented interactions and proteins.

**Table 5:** Available resources, databases and tools to retrieve and analyze plant PPI data.

**Resource**	**Discription**	**URL**	**References**
**ARAPORT**
The Arabidopsis information portal	Open-access online community resource for Arabidopsis research	https://araport-dev.tacc.utexas.edu/	Krishnakumar et al., 2015
**AraraPPINet**
The global Arabidopsis PPI network	Genome-wide Arabidopsis PPI network inferred from known 3D structures and functional evidence	http://netbio.sjtu.edu.cn/arappinet/	Zhang et al., 2016
**AtPID**
The *Arabidopsis thaliana* Protein Interactome Database	Integrative database for Arabidopsis PPI data and function annotations, based on prediction methods and literature	http://www.megabionet.org/atpid/webfile/	Cui et al., 2008
**AtPIN**
The *Arabidopsis thaliana* Protein Interaction Network	Database and web interface for searching and building interaction networks based on publicly available PPI datasets	https://atpin.bioinfoguy.net/cgi-bin/atpin.pl	Brandão et al., 2009
**AtTFIN-1**
The *Arabidopsis thaliana* transcription factor interaction network	Interactive web interface for the deep-coverage Arabidopsis transcription factor interactome	http://signal.salk.edu/interactome/AtTFIN-1.html	Trigg et al., 2017
**BAR**
The Arabidopsis interactions viewer	Web interface with predicted and confirmed Arabidopsis PPIs and functional information, based on various literature sources	https://bar.utoronto.ca/interactions/cgi-bin/arabidopsis_interactions_viewer.cgi	Geisler-Lee et al., 2007
**BioGRID**
The Biological General Repository for Interaction Dataset	Database of curated physical and genetic interactions, chemical associations and post-translational modifications (PTMs)	https://thebiogrid.org/	Chatr-Aryamontri et al., 2017
**CORNET**
CORelation NETworks in plants	Online tool for easy access to Arabidopsis and Maize PPIs, co-expression and regulatory data	https://bioinformatics.psb.ugent.be/cornet/	De Bodt et al., 2012
**Cytoscape**
Network visualization software	Open source software for network visualization, analysis and integration of additional data	http://www.cytoscape.org/	Bauer-Mehren, 2013
**InACT**
The molecular interactions database	Open source database system and analysis tools for molecular interaction data, derived from literature curation or direct user submissions	http://www.ebi.ac.uk/intact/	Orchard et al., 2014
**iPfam**
The protein family interaction database	Database of protein family and domain interactions, based on known 3D structures	http://xfam.org/	Finn et al., 2014
**MINT**
The Molecular INTeraction database	Integrative database with experimentally verified PPIs from curated literature	http://mint.bio.uniroma2.it/	Licata et al., 2012
**PAIR**
The Predicted Arabidopsis Interactome Resource	Database system and network analysis tools for predicted Arabidopsis PPIs	http://www.cls.zju.edu.cn/pair/	Lin et al., 2011
**PPIM**
The Protein-Protein Interaction database for Maize	Comprehensive database with physical, functional and molecular interactions from literature and public databases	http://comp-sysbio.org/ppim/	Zhu et al., 2016
**PPIN-1**
The Plant-Pathogen Immune Network 1	Interactive web interface for the first plant-pathogen immune network	http://signal.salk.edu/interactome/PPIN1.html	Arabidopsis Interactome Mapping Consortium, 2011
**PRIN**
The Predicted Rice Interactome Network	Integrative database with rice PPIs based on predicted (interolog) interactions	http://bis.zju.edu.cn/prin/	Gu et al., 2011
**PTIR**
The Predicted Tomato Interactome Resource	Integrative database with tomato PPIs based on predicted (interolog) interactions	http://bdg.hfut.edu.cn/ptir/index.html	Yue et al., 2016
**RicePPINet**
The Rice PPI Network	Genome-wide rice PPI network inferred from structural relationship and functional information	http://netbio.sjtu.edu.cn/riceppinet	Liu et al., 2017
**STRING**
Protein-protein Interaction Networks	Database of physical and functional PPIs, inferred from computational predictions, knowledge transfer between organisms and data from other databases	https://string-db.org/	Szklarczyk et al., 2017
**TAIR**
The Arabidopsis Information Resource	Database of Arabidopsis genetic and molecular biology data	https://www.arabidopsis.org/	Berardini et al., 2015

## Hubs in large-scale plant PPI networks: the lack of overlap

In general, hub proteins are most often identified when studying large-scale protein networks. Table 2 gives a historical overview of large-scale plant PPI studies resulting in networks containing at least 100 proteins, with first reports starting in 2006. As illustrated, they consist of both broad-spectrum as well as more specific studies, mainly focusing on the *A. thaliana* interactome and primarily on signal transduction, stress responses, cell proliferation, protein ubiquitination, transcription factors, and membrane-protein interactions. The importance of hubs in these networks cannot be underestimated, however, less than half of the large-scale plant PPI studies focus on or even mention the identification of hub proteins (Table 2). Moreover, within those studies hubs are often defined differently and/or focus is restricted to hubs within a specific protein (sub)network of interest. Also apparent, both identified PPIs as well as the total amount of interactions (degree) found for specific proteins significantly differ between studies, especially when comparing computational and experimental networks. As a consequence, the overlap in plant proteins labeled as hubs in different studies is small and the differences in degree and characteristics between the identified hubs once more illustrate that there is no real unifying and binding definition for PPI network hubs (Tables 3, 4, Supplementary Tables 1, 2).

### Computationally predicted plant hubs

The first real large-scale plant PPI network was computationally predicted for *A. thaliana* through interolog mapping, predicting 19,979 interactions for 3,617 *A. thaliana* proteins (Geisler-Lee et al., 2007). Hub proteins of different degrees were identified, distinguishing between minor (3–5 interactions), small (6–10 interactions), medium (11–50 interactions), major (51–100 interactions), and super hubs (101+ interactions). In general, proteins had an average of 11 interactors with the largest class consisting of the medium hubs (Table 3). This same study showed that major and super hubs tend to have nuclear localization, with 11% of them annotated as indispensable. The most highly connected hubs appeared to be highly conserved proteins, involved in important signaling processes (Geisler-Lee et al., 2007). Later, hubs were also identified via similar interolog-based computational studies for *Coffea canephora* (Geisler and Fitzek, 2011), *Oryza sativa* (Gu et al., 2011; Ho et al., 2012), *Brassica rapa* (Yang et al., 2012), *Zea mays* (Musungu et al., 2015), *Physcomitrella patens* (Schuette et al., 2015), *S. lycopersicum* (Yue et al., 2016), and *Macrotyloma uniflorum* (Bhardwaj et al., 2016). In these studies a higher number of reference species was used as more interactome data became available (Table 3), including a diverse range of eukaryotic and prokaryotic organisms, allowing greater confidence (Schuette et al., 2015). An exception was the *B. rapa* interactome, inferred only from PPI data of *A. thaliana*, belonging to the same family of *Brassicaceae* (Yang et al., 2012).

Differences can be seen between the average amount of interaction partners and the largest hub class inferred from these interactomes (Table 3), but similar general conclusions regarding the hub proteins were made. As in the *A. thaliana* study, the highest degree hubs tend to be highly conserved and essential proteins, including heat shock proteins, nuclear DNA repair proteins, cytoskeleton-related proteins, ribosomal proteins and proteins associated with ubiquitin-mediated breakdown pathways (Supplementary Table 2; Geisler-Lee et al., 2007). However, conserved hub proteins with an unknown function were also always identified. More information on these computational plant interactome studies can be found in the corresponding publications (Geisler-Lee et al., 2007; Geisler and Fitzek, 2011; Gu et al., 2011; Ho et al., 2012; Yang et al., 2013; Musungu et al., 2015; Schuette et al., 2015; Bhardwaj et al., 2016; Yue et al., 2016).

### Experimentally determined plant hubs

The first experimentally constructed and comprehensive large-scale plant interactome was presented by the Arabidopsis Interactome Mapping Consortium (2011). This *A. thaliana* interactome (Arabidopsis Interactome 1, AI-1) was constructed using a systematic binary Y2H-based approach screening the pairwise interactions between 8,543 *A. thaliana* proteins (constructed from AtORFeome2.0) and resulted in the identification of 5,664 highly reliable interactions among 2,661 proteins (Arabidopsis Interactome Mapping Consortium, 2011). A subsequent study using the same Y2H-based approach determined the interactions between the 8,543 initially screened *Arabidopsis* proteins (in AI-1) and an extra 552 *A. thaliana* immunity proteins and effector proteins from the phytopathogens *Pseudomonas syringae* and *Hyaloperonospora arabidopsidis*. This resulted in an extended PPI network composed of 1,358 interactions among 926 proteins, including 83 pathogen effectors, 170 immune proteins, and 673 other *A. thaliana* proteins. Combining this network with AI-1 and literature-curated interactions for the 926 interacting proteins, resulted in a plant-pathogen immune network (PPIN-1), containing 3,148 interactions (Mukhtar et al., 2011). Additionally, 15 *A. thaliana* proteins with a degree higher than 50 in AI-1 were identified as hub proteins (hubs_50_), with 7 of these hubs_50_ targeted by effectors from both *P. syringae* and *H. arabidopsidis* (Supplementary Table 1). A third study defined the interactions between 12,000 *A. thaliana* proteins, including the 8,543 initially screened proteins, and pathogen effectors from *Golovinomyces orontii*, yielding a *G. orontii* effector-host interactome network (*Gor*_EHIn_12k_). Combining all *P. syringae, H. arabidopsidis*, and *G. orontii* effector host-protein interactions with interaction data from AI-1, PPIN-1, and literature, yielded the comprehensive Plant-Pathogen Immune Network 2 (PPIN-2; Weβling et al., 2014). When limited to the interactions of the 8,543 initially screened proteins, a network was generated with identical experimental parameters consisting of 178 *A. thaliana* host proteins and 123 effectors, connected by 421 effector host-proteins interactions and 162 interactions among host proteins (PPIN-2_8k_sys_) (Weβling et al., 2014). These studies allowed the observation of a remarkable convergence of effectors onto a common set of host hub proteins, including members of the TCP (teosinte-branched/cycloidea/proliferating cell factor) TF family and the LSU (low sulfur upregulated) protein family (Supplementary Table 1). Similarly to the computational studies, also some proteins with an unknown function were identified as hubs.

Additionally, several more specific large-scale experimental studies identifying hub proteins in *A. thaliana* were published. These include a protein microarray study focusing on calmodulin targets (Popescu et al., 2007) and Y2H studies focusing specifically on the TOPLESS (TPL) transcriptional co-repressor (Causier et al., 2012) and ABA (abscisic acid) (Lumba et al., 2014; Table 4). Though multiple hubs were identified in these studies, they were defined as highly (inter)connected clusters of proteins in the unraveled networks and not as single hub proteins. More recently, the newly developed CrY2H-seq method, allowed the creation of a deep-coverage *A. thaliana* TF interaction network (AtTFIN-1), greatly expanding the number of known TF interactions and providing more evidence for the validation of TCP TFs as hub proteins (Trigg et al., 2017). One large-scale *A. thaliana* interactome study focused on membrane protein interactions using sUbq-approaches and identified several membrane hub proteins. A test-space of 6.4 × 10^∧^6 protein pairs was covered, determining 12,102 membrane/signaling PPIs, with more than 99% of the interactions previously unknown. Interactions were confirmed at a rate of 32% in *in planta* split–GFP interaction assays (Jones et al., 2014). A total of 46 hub proteins with a degree higher than 70 were identified, with functions consistent with roles that require interaction with a large number of target proteins (Table 4; Jones et al., 2014). However, as membrane PPIs require specific approaches to be identified and are generally not picked up by other methods, the unraveled plant membrane protein interactome is even more incomplete and many biologically relevant interactions are undoubtedly still being missed.

Aside from *A. thaliana*, several experimental large-scale interactome studies have also been published for economically important crops, but only one Y2H study for *O. sativa* focused on hub proteins, with several identified as having a key role in the stress response (Table 4; Seo et al., 2011).

### Other plant hub-defining studies

To be complete, next to extended interactome studies, some small-scale PPI studies also define hubs in their limited (sub)networks, but these can hardly be seen as true hubs. Additionally, several other reports define plant hub proteins primarily based on their central role in signaling pathways. When it is indeed shown through PPI studies that these proteins engage in numerous physical interactions with other proteins, these can be correctly labeled as true hub proteins. By contrast, when they are solely termed hubs because of their importance in signaling, one could seriously doubt this labeling, based on the original hub protein definition. Although, this could also be interpreted as a slow emergence of a distinction between degree hubs (based on physical connectivity) and signaling hubs (based on a central signaling role). The fact that some hubs fall into both categories, however, and the distinction between the categories is not clear to all, often causes confusion for researchers from different fields, e.g., functional biologists vs. network theorists.

Proteins that are often labeled as signaling hubs include various transcription factors with a role in the plant hormone response, including ERFs (Ethylene response factors), ARFs (Auxine response factors), TPL (TOPLESS), and JAZ (Jasmonate ZIM-domain) proteins (Sheard et al., 2010; Mukhtar et al., 2011; Wager and Browse, 2012; Piya et al., 2014; Windram et al., 2014; Müller and Munné-Bosch, 2015), various PP2Cs (protein phosphatase 2C) and MAPKs (Mitogen-activated protein kinases) (Pitzschke et al., 2009; Dietz et al., 2010; Rodrigues et al., 2013), heat shock proteins (Taipale et al., 2010), and several ubiquitin ligase complex-associated proteins (Shabek and Zheng, 2014). Others identified in more focused studies include PIF (Phytochrome Interacting Factor) proteins (Leivar and Quail, 2011; Pfeiffer et al., 2014), peroxiredoxins (Muthuramalingam et al., 2009), calcium-dependent protein kinases (Schulz et al., 2013; Ranty et al., 2016), GTPases (Dietz et al., 2010), and specific proteins like Mediator 25 (Çevik et al., 2012), RPM1-interacting protein 4 (Sun et al., 2014), the miR156/SPL module (Wang and Wang, 2015), and the stress-associated plant protein RCD1 (radical-induced cell death 1; O'Shea et al., 2017).

## Plant stress response hubs: an overview of the major classes

Many plant proteins labeled as hubs play a role in the plant stress response. Most of these are true hubs, participating in numerous PPIs and having an important central role in signaling pathways. They broadly fall into the functional categories of transcription factors, kinases and phosphatases, or components of ubiquitin ligase complexes. Other hubs include heat shock proteins, proteins involved in redox signaling, DNA repair, ribosomal proteins, and cytoskeleton-interacting proteins that play a role in protecting cells and cell components, making them essential and often conserved. There are, however, also proteins labeled as highly interacting hubs which are still uncharacterized or do not have a completely unraveled function, making them interesting targets for future research. In the next paragraphs we will discuss some of the best studied plant hub representatives with a role in the plant stress response, specifically focusing on hub protein characteristics. The resulting findings are summarized in Table 6.

**Table 6:** Key features of the major classes of plant stress response-related hub proteins.

**Hub class**	**Function**	**Connectivity**	**Structure**
Transcription factor hubs e.g., JAZ3	Gene expression regulation in response to the environment, often central in plant hormone regulatory networks	Highly (inter)connected proteins, mostly co-expressed with their interactors that regulate their activity, localization and abundance	Often multiple distinguishing and conserved domains for DNA and protein binding and significant intrinsic disorder
Kinase and phosphatase hubs e.g., ABI1	Protein (de)phosphorylation mediating stress signal translation, amplification, modification and integration	Highly (inter)connected proteins that bind specific targets at specific times and locations in response to specific stimuli	Ordered proteins with specific binding domains and little intrinsic disorder, but flexible hinges and linker regions
Ubiquitin system associated hubs e.g., SUMO1	Targeted protein degradation or regulation of protein localization, structure, function and interaction capability	Highly connected proteins that bind numerous targets, mostly determined as hubs in computational studies	Varying due to protein diversity, often conserved regions and domains
Chaperone and co-chaperone hubs e.g., HSP90	Protein stabilization, refolding and prevention of aggregation during stress conditions	Highly (inter)connected proteins with a constantly varying degree of binding, depending on the situation and localization	Varying due to protein diversity, often form dimers and have tetratricopeptide (TPR) regions to facilitate PPIs
Redox signaling hubs e.g., TRX5	Regulation of complex redox networks, mediating electron transport and distribution	Highly (inter)connected redox network proteins with a large set of cellular protein targets for electron transport	Small proteins varying from largely unstructured to having a high degree of secondary structure and stability
Functionally unclear hubs e.g., LSU1	Unknown or not completely unraveled function with evidence for a role in stress responses	Highly (inter)connected proteins, identified as top hubs with unknown function in PPI networks	Varying due to protein diversity, mostly unknown or resembling certain protein family structures, usually flexible regions

### Transcription factor hubs

**Figure 3:**
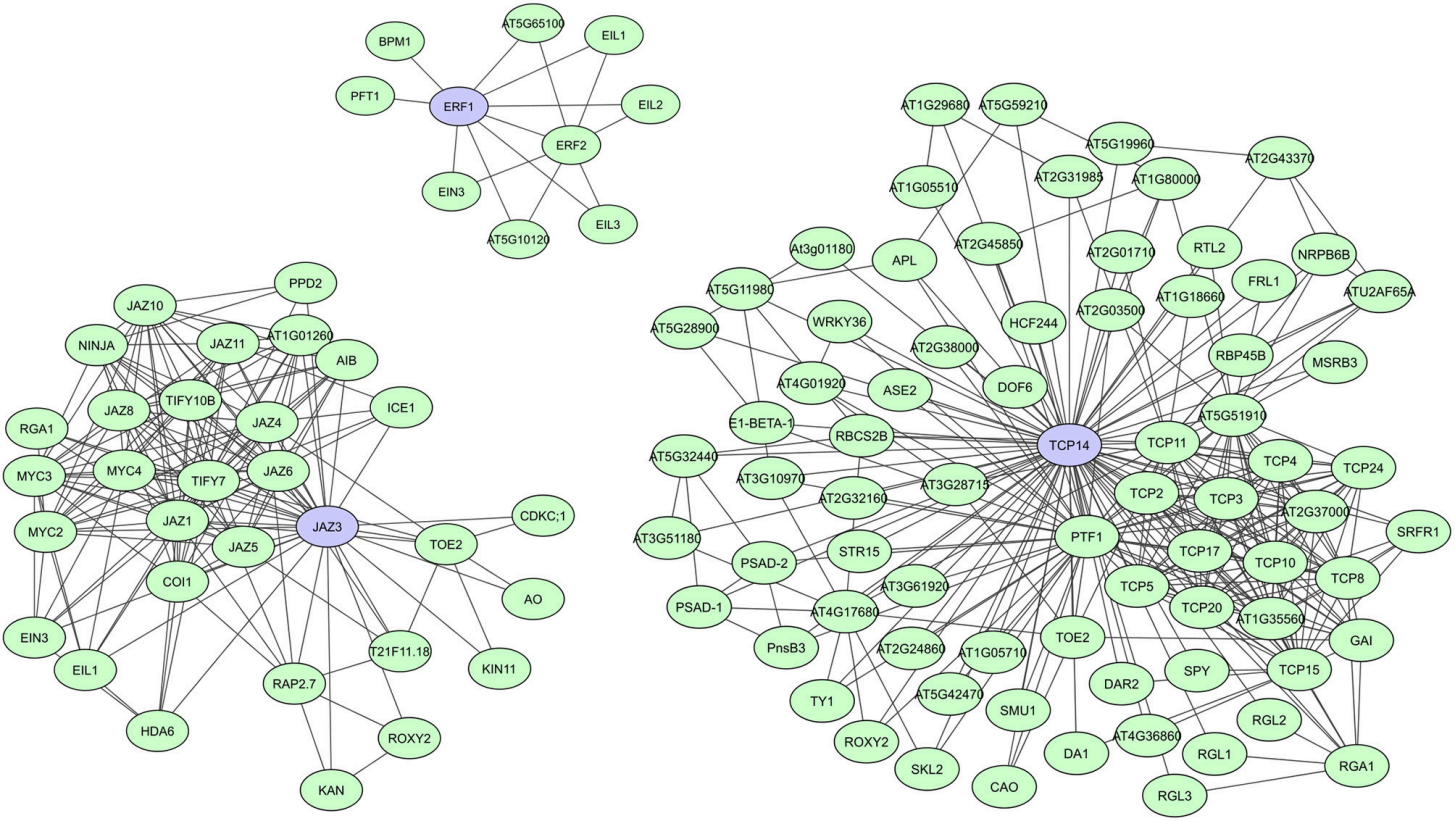
STRING-based networks for the *A. thaliana* ERF1, JAZ3, and TCP14 transcription factors. Interactors experimentally determined or from curated databases (medium confidence 0.400).

During evolution, DNA-binding proteins became prevalent in eukaryotic genomes and several families of transcription factors (TFs) arose. These proteins contain specific domains for DNA-binding, but their activity, localization, and abundance is often regulated through binding with other proteins. TFs usually have multiple conserved DNA and protein binding domains, but studies also show a significant prevalence of intrinsic disorder in eukaryotic TFs, making them more flexible and efficient (Liu et al., 2006). Recently published *A. thaliana* TF interactome networks show a large amount of interactions for most TF (Yazaki et al., 2016; Trigg et al., 2017). In the network created by Yazaki et al. (2016), on average 95 interactors were found, with a maximum of 499 interactors reported for TGA1, a bZIP TF important in auxin and SA signaling (Yazaki et al., 2016). Other studies specifically indicated several plant TFs as hubs regulating transcriptional cascades, including *A. thaliana* JAZ proteins, ERFs, ARFs, TCPs, and TPLs (Mukhtar et al., 2011; Pauwels and Goossens, 2011; Causier et al., 2012; Piya et al., 2014; Li et al., 2015; Müller and Munné-Bosch, 2015; Trigg et al., 2017). JAZ proteins, ERFs, and TCPs all have important roles in plant stress responses and defense response pathways. JAZ family proteins function as transcriptional repressors of jasmonate (JA) signaling (Pauwels and Goossens, 2011; Kazan and Manners, 2012; Wager and Browse, 2012), while ERFs regulate molecular responses to both pathogens and abiotic stresses (Mizoi et al., 2012; Müller and Munné-Bosch, 2015). TCPs regulate plant growth and development through control of cell proliferation and differentiation (Aguilar-Martínez and Sinha, 2013), but have recently also been linked to plant immunity (Sugio et al., 2011; Kim et al., 2014; Weβling et al., 2014). In comparison to JAZ and TCP TFs, however, only a limited number of interactions are currently known for ERFs (Figure 3).

Most JAZ family proteins are highly connected and interconnected and share many of their interactors with which they are co-expressed (Pauwels and Goossens, 2011). Typical JAZ interactors are DELLA proteins, COI1 (CORONATINE INSENSITIVE1) and NINJA, but they also form homo- and heterodimers and interact with several other TFs (Figure 3) (Pauwels and Goossens, 2011; Wager and Browse, 2012). *A. thaliana* JAZ3, for example, was shown to have 23 interactors in AI-1 and is targeted by effectors of three distinct pathogens (Supplementary Table 1) (Arabidopsis Interactome Mapping Consortium, 2011; Mukhtar et al., 2011; Weβling et al., 2014). Currently over 30 interactors have been determined for JAZ3 according to the STRING database (Figure 3). JAZ mutants also show clear effects on pathogen susceptibility, metabolism, and development (Pauwels and Goossens, 2011; Pieterse et al., 2014; Weβling et al., 2014). For the TCP TF family it seemed at first that they could have highly connected and interconnected members, as well as much less connected members. As such, *A. thaliana* TCP14 and TCP13 were characterized as hub_50_ proteins in AI-1 and as targets of three distinct pathogens, while TCP15 and TCP19 were shown to have over 20 interactors and be targets of at least two pathogens, and TCP1 was only found to have two interactors (Supplementary Table 1; Arabidopsis Interactome Mapping Consortium, 2011; Mukhtar et al., 2011; Weβling et al., 2014). However, the most recently created *A. thaliana* TF interactome network (AtTFIN-1) expanded the number of known TCP TF interactions. TCPs were shown to interact with 18 other TF families and although differences in degree between TCP family members were still apparent, with over 200 interactors found for TCP14 compared to 30 interactors for TCP1, most were found to be highly interacting (Trigg et al., 2017). Additionally, several TCP mutants were shown to have altered disease susceptibility phenotypes, suggesting an important and universal role of this class of TFs during pathogen infection (Weβling et al., 2014).

### Protein kinase and phosphatase hubs

Plant signaling pathways are highly regulated through reversible protein phosphorylation, mediated by protein kinases and phosphatases (Chae et al., 2010). Some function in integrating signals triggered by a wide range of stresses and are thus said to function as key hubs. These include certain calcium-dependent protein kinases (CDPKs), calmoduline (CaM)-like proteins (CMLs), mitogen-activated protein kinases (MAPKs), and serine/threonine proteins phosphatases (including PP2Cs).

CDPKs comprise Ser/Thr protein kinases with a conserved structure and several ordered domains. They function as complex signaling date hubs, able to interact with many different proteins, but with specific targets at specific times and locations, in response to specific environmental stimuli (Schulz et al., 2013). Plant CaMs and CMLs have a wide range of diverse targets, including TFs, intracellular and receptor protein kinases, F-box proteins, RNA-binding proteins, and several proteins of unknown function. They have little disorder in their structure, but highly flexible hinges and linker regions that allow for the binding with multiple partners (Popescu et al., 2007). Several *A. thaliana* CaM/CML proteins, including CaM1, CaM6, CaM7, and CaM9, were shown to be very highly connected and form the main hubs of the CaM/CML network (Table 4, Popescu et al., 2007).

MAPK signaling cascades are often required for further translation, amplification and modification of environmental stimuli. All MAPKs share similar two lobed 3D structures, with a protein substrate binding on the surface of the C-terminal domain and the phosphorylation loop sequence influencing substrate specificity. However, MAPK signaling location, specificity and duration are regulated by interacting scaffold proteins and MAPK phosphatases (Taj et al., 2010). Both MPK3 and MPK6, well-known MAPKs in plant defense responses (Beckers et al., 2009), are said to function as date hubs (Dietz et al., 2010). Using a protein microarray-based method allowing high-throughput study of protein phosphorylation, 48 and 39 potential substrates could be identified for MPK3 and MPK6, respectively (Feilner et al., 2005). Later, a study of Popescu et al. (2009), focusing on MAPK targets (Table 2) found that each MAPK bound and phosphorylated an average of 128 other proteins, with 184 phosphorylated by MPK6 (Popescu et al., 2009).

**Figure 4:**
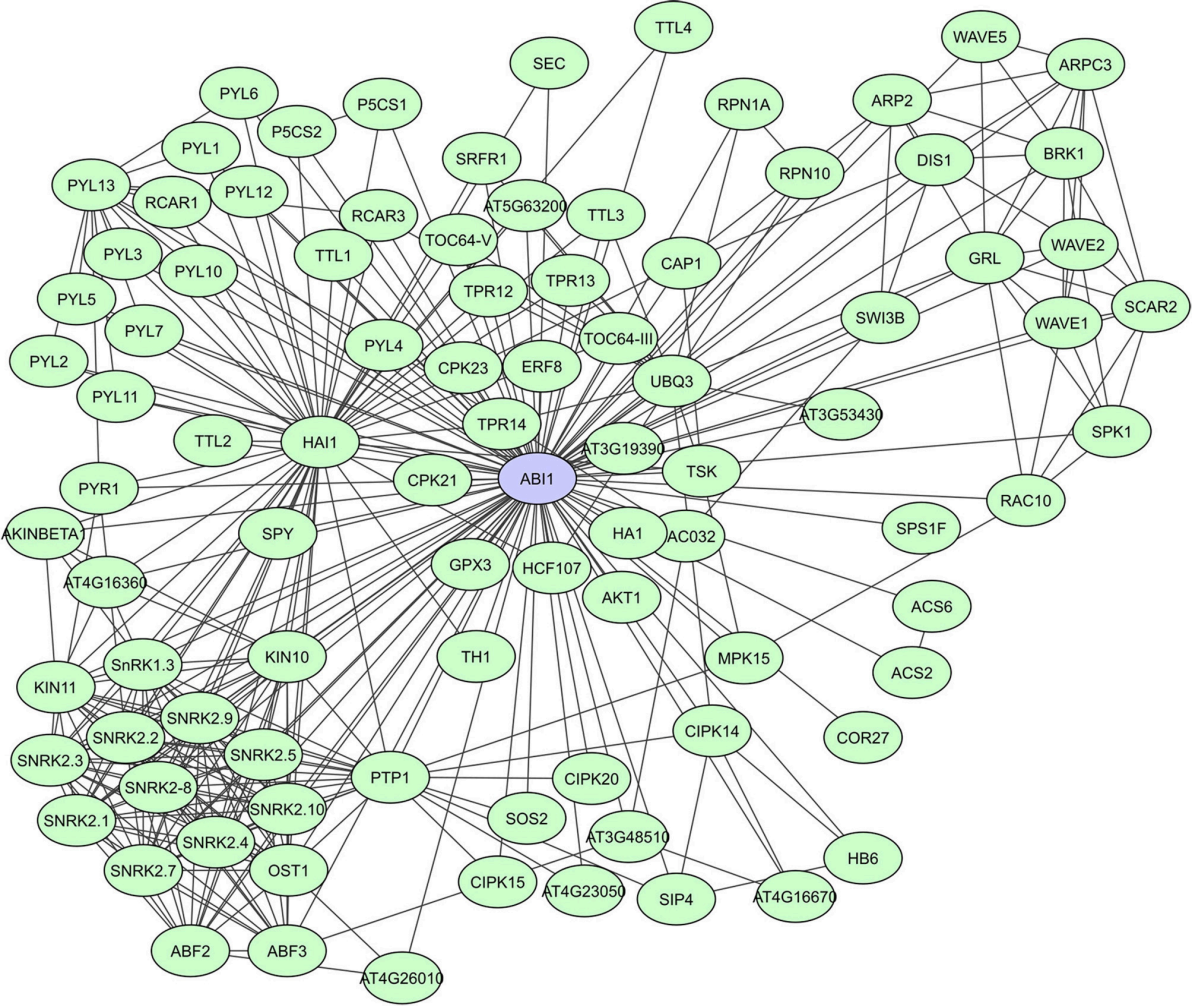
STRING-based network for the *A. thaliana* ABI1 protein phosphatase. Interactors experimentally determined or from curated databases (medium confidence 0.400).

PP2C-type protein phosphatases are involved in signaling regulation, cooperating with other phosphatases and kinases, often in a stress-induced manner (Rodriguez, 1998; Sheen, 1998; Merlot et al., 2001). A general characteristic of PP2C-type phosphatases is the presence of 11 subdomains in the catalytic part of the proteins. PP2Cs interact with both substrates as well as regulator proteins, via N-terminal extensions that function as binding sites to specific substrates (Schweighofer et al., 2004). In *A. thaliana*, clade A PP2Cs act as regulatory hubs for different abiotic stress responses via interaction with a wide array of targets. They function as negative regulators of the abscisic acid (ABA) signaling pathway through their interaction with SnRK2s [SNF1 (Sucrose Non-fermenting-1) -Related Protein Kinase 2]. The PP2Cs ABI1 and PP2CA, were shown to also function as SnRK1 phosphatases (Rodrigues et al., 2013). A STRING-based interaction network for ABI1 shows more than 90 determined interactors, including many other phosphatases and kinases, TFs, metabolic enzymes and proteins of unknown function (Figure 4). Lastly, the PP2C HAI1, was indicated as a ABA signaling interactome hub, highly correlated with the expression of many of its partners in both osmotic and salt stress data sets (Lumba et al., 2014).

### Ubiquitin system associated hubs

The ubiquitin (Ub)-proteasome protein breakdown system has a fundamental role in plants, both in growth and development but also in plant immune signaling (Stone and Callis, 2007; Delauré et al., 2008; Marino et al., 2012). Ubiquitination or ubiquitin-like modifications, such as sumoylation, can regulate plant stress signaling via affecting protein localization, structure, function, and interaction capability (Miura and Hasegawa, 2010). About 6% of the plant proteome is said to be composed of Ub-proteasome system (UPS) related proteins (Hua and Vierstra, 2011).

**Figure 5:**
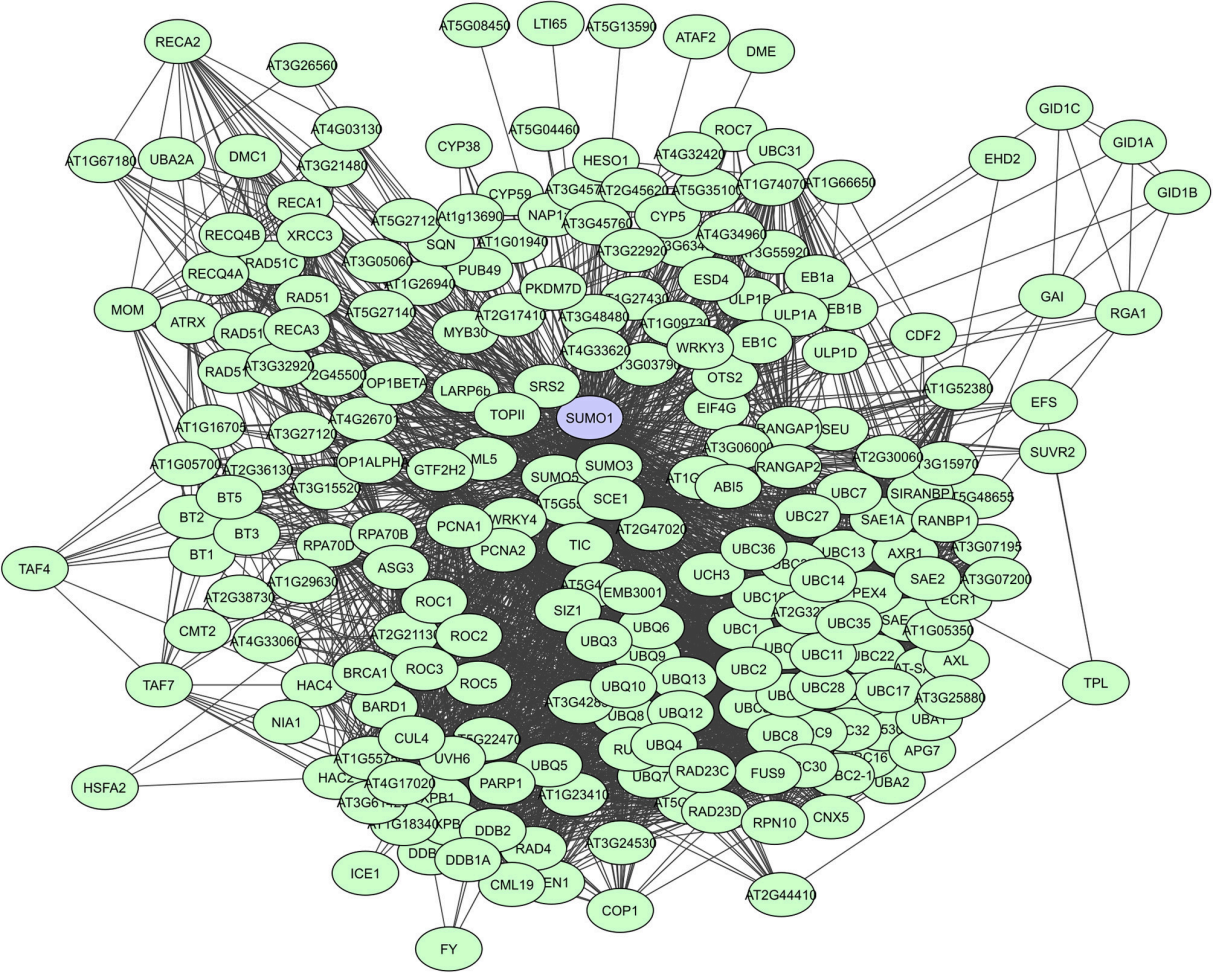
STRING-based network for the *A. thaliana* Ub-like modifier SUMO1. Interactors experimentally determined or from curated databases (medium confidence 0.400).

When looking at large-scale plant PPI studies, UPS proteins are especially identified as highly interacting proteins in computational studies. In the Geisler-Lee study (2007) the small Ub-like modifier AtSUMO1 was identified as the top hub with 172 interactors, followed by the Ub carrier protein AtUBC1 with 119 interactors, and the multi-Ub chain binding 26S proteasome subunit protein ATMCB1 with 108 interactors (Supplementary Table 2). Many AtSUMO1 interactions have however also been confirmed via a Y2H screen (Figure 5, Elrouby and Coupland, 2010). In later computational PPI studies for *C. canephora* (Geisler and Fitzek, 2011) and *Z. mays* (Musungu et al., 2015) UPS proteins were also found as top hubs (Table 3). In the experimental membrane-linked interactome composed by Jones et al. (2014) several UPS proteins were identified as hub proteins with degrees higher than 70 (Jones et al., 2014). Additionally, the F-box proteins or substrate receptors of multisubunit E3 ligase complexes, like the plant Skp-cullin-F-box complexes (SCF) and the cullin-RING ligases (CRLs) are often considered as hub proteins (Shabek and Zheng, 2014). A typical example is the *A. thaliana* COI1 F-box protein that allows the SCF^COI1^ complex to regulate JA-responsive gene expression through JAZ protein breakdown (Pauwels and Goossens, 2011). Still, UPS proteins are seemingly less prevalent in experimentally determined interactomes. This is probably a result of the more specific nature of some of these interactomes and because general UPS proteins are often put on non-specific interactors lists as they are almost always present and often found to interact with many proteins.

### Chaperone and co-chaperone hubs

Chaperone proteins or heat shock proteins (HSPs) are essential for cellular homeostasis and are responsible for proper protein folding, localization, and degradation (Wang et al., 2004; Al-Whaibi, 2011). Five major families of plant chaperones are currently recognized according to their approximate molecular weight: the HSP70 family, the chaperonins, the HSP90 family, the HSP100 family and the small HSP (sHSP) family (Gupta et al., 2010). As a result of their stabilizing function they interact with numerous substrates, but also with several co-chaperones that regulate their activity, substrate recognition and refolding (Al-Whaibi, 2011; Breiman, 2014). As a result, both chaperones and co-chaperones are often identified as hubs.

**Figure 6:**
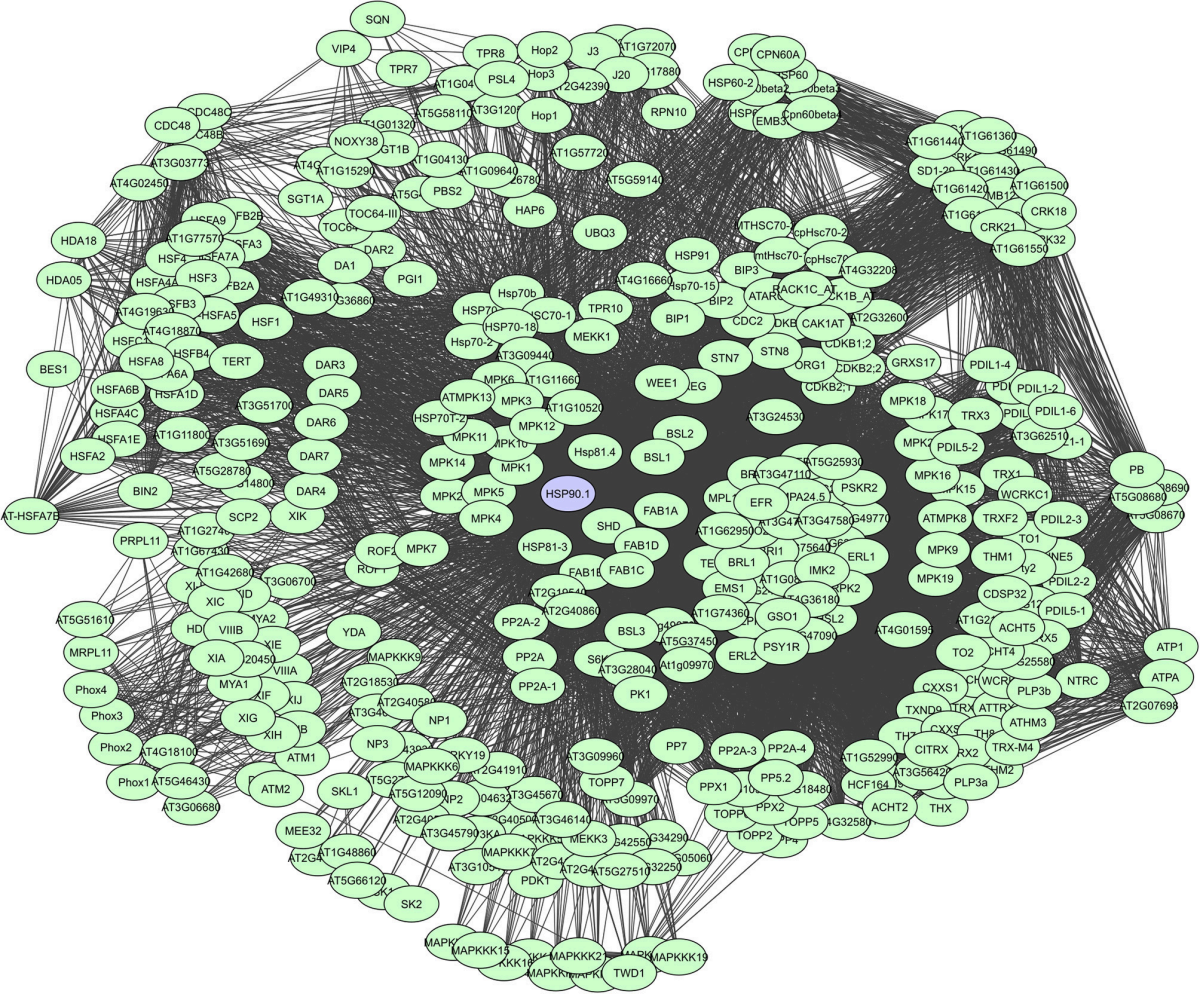
STRING-based network for the *A. thaliana* HSP90.1 chaperone. Interactors experimentally determined or from curated databases (medium confidence 0.400).

As such, HSP90s, for example, are core components of many protein complexes and act as key regulators of plant growth and immunity, by directly interacting with R proteins and diverse proteins like kinases and TFs that activate defense responses (Liu et al., 2004; Sangster and Queitsch, 2005; Sangster et al., 2007; Breiman, 2014; Park and Seo, 2015). They are large dimeric proteins with each monomer having three characterizing domains i.e. a highly conserved N-terminal domain that binds and hydrolyzes ATP following substrate interaction, a middle domain thought to have an important role in substrate recognition, and a C-terminal domain mediating dimerization and interaction with many co-chaperones (Taipale et al., 2010). Chaperone proteins also often have tetratricopeptide regions (TPRs) which can form scaffolds to mediate PPIs or the assembly of large protein complexes (Blatch and Lässle, 1999). However, their binding degree varies continuously depending on the cell and tissue type, interactions with other proteins, alternative splicing, post-translational modifications and cell signaling events. A total of more than 380 interactors have currently been determined for HSP90.1, based on the STRING database (Figure 6).

Another example is the mitochondrion-localized small heat shock protein AtHSP23.6 that was identified as one of the hub_50_ proteins in AI-1, with 87 interactors, and targeted by *H. arabidopsidis* effector proteins (Supplementary Table 1; Arabidopsis Interactome Mapping Consortium, 2011; Mukhtar et al., 2011). In the studies of Ho et al. (2012) and Yue et al. (2016) a rice HSP90 and a tomato HSP70 were found as top hub proteins with 686 and 3,7551 interactions, respectively (Table 3; Ho et al., 2012; Yue et al., 2016). In the computational study of Geisler-Lee et al. (2007) the prefoldin co-chaperone AtPDF6 was identified as one of the top 20 hub proteins with 93 predicted interactors. However, only five of these interactors were experimentally confirmed in AI-1 (Supplementary Table 2; Arabidopsis Interactome Mapping Consortium, 2011; Mukhtar et al., 2011). Like UPS proteins, chaperones, and co-chaperones, are seemingly less prevalent in datasets from experimentally determined plant interactomes. This can be the result of HSPs not covalently but transiently binding to their targets, not making them part of the final complex and thus being more difficult to pick up with certain experimental approaches. HSPs are also often not included in more specific screens or are often added to lists of false positive or non-specific interactors as they are inherently associated with many proteins as a result of their stabilizing function.

### Redox signaling hubs

Reactive oxygen species (ROS) in plants are produced through mitochondrial respiration and photosynthetic electron transport in the chloroplasts (Foyer and Noctor, 2013; Schwarzländer and Finkemeier, 2013; Kalia et al., 2017). Regulating ROS and redox signals is essential for plant survival and requires complex redox networks. These networks contain several small hub proteins, including certain ferredoxins, thioredoxins, and peroxiredoxins, that play a central role in electron distribution by mediating electron transfer through interacting with many other redox network proteins (Dietz, 2008; Dietz et al., 2010).

**Figure 7:**
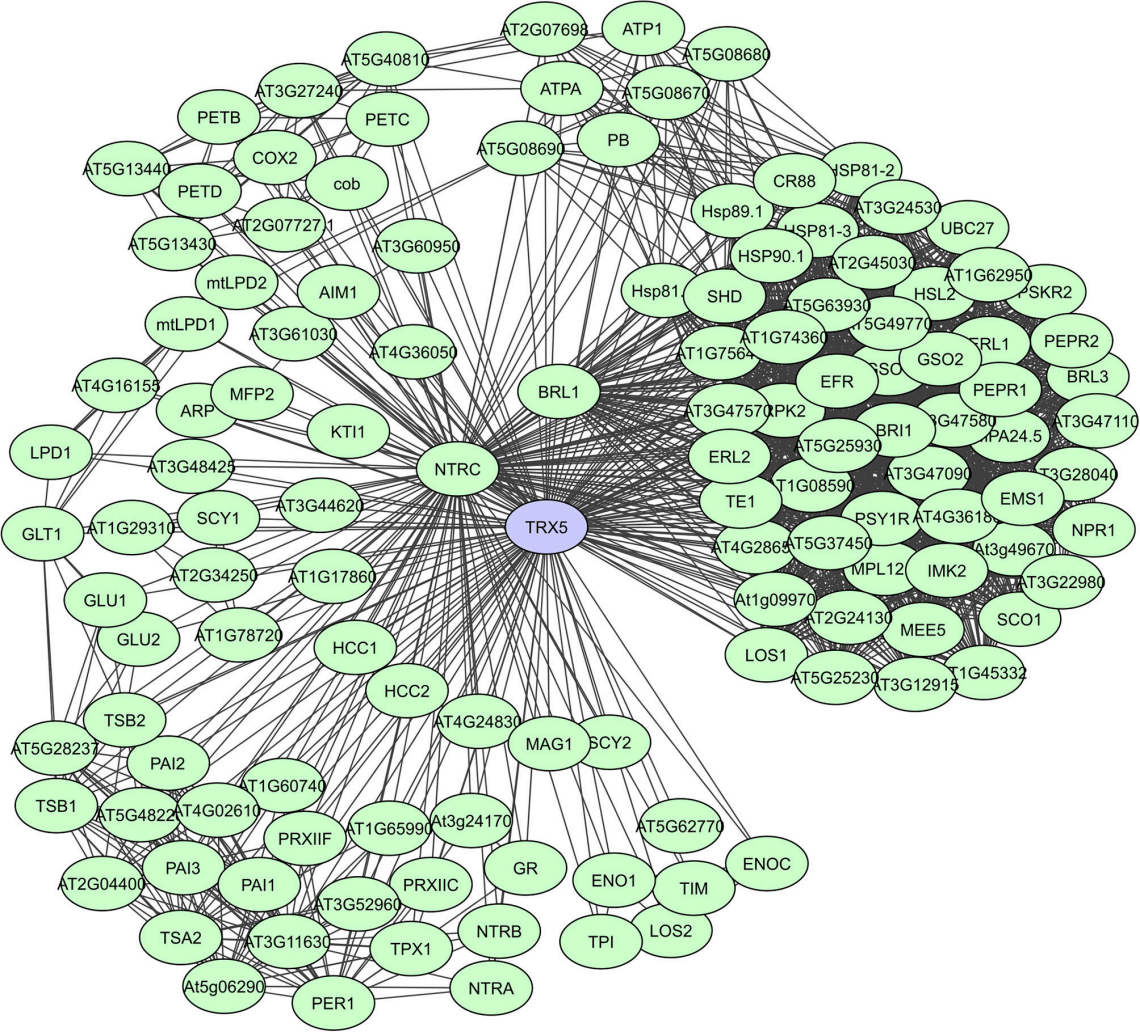
STRING-based networks for the *A. thaliana* TRX5 thioredoxin. Interactors experimentally determined or from curated databases (medium confidence 0.400).

Fe_2_S_2_ ferredoxins in chloroplasts function as electron carriers in the photosynthetic electron transport chain and as electron donors to various cellular proteins. They have a large proportion of unstructured regions with a high content of loops, but the residues necessary for PPIs are present in the α-helices (Dietz et al., 2010). Thioredoxins (Trx) also accept electrons through thiol-disulfide interchange and adjust the redox state of multiple target proteins. They have a high degree of secondary structure coupled to an extraordinary stability. In plants, the Trx system is particularly complex and many isoforms exist in various subcellular compartments, with a large set of plant Trx targets, exceeding 300 proteins, having been identified (Motohashi et al., 2001; Marchand et al., 2004; Lemaire et al., 2007; Meyer et al., 2009). In the experimental membrane-linked interactome composed by Jones et al. (2014) the *A. thaliana* H-type Trx ATH7 and ATH5 were identified as hub proteins with 97 and 71 interactors, respectively. ATH5 seems to be specifically involved in responses to pathogens and oxidative stress, and also exhibits antimicrobial activity (Laloi et al., 2004; Park et al., 2017). A STRING-based interaction network for ATH5 (TRX5) shows more than 120 interactors (Figure 7). Lastly, peroxiredoxins function as thioredoxin-dependent peroxidases and in plants 2-CysPyr functions to protect the photosynthetic membrane against photo-oxidative damage (Baier and Dietz, 1999). In the computational interactome of Geisler-Lee et al. (2007) 52 interactors were identified for 2-CysPyr. Multiple redox and non-redox interactions define 2-CysPyr as a regulatory hub in the chloroplast (Muthuramalingam et al., 2009; Dietz et al., 2010).

### Functionally unclear hubs

In many of the large-scale plant interactome studies, several putative proteins have been identified as hubs, like the unknown hub_50_ proteins in AI-1 (Supplementary Table 1), or proteins with a not yet completely unraveled function. A typical example of the latter is the family of LSU proteins, identified as hubs with a potentially important but not (fully) understood function in the plant stress response. These small proteins were initially identified as strongly induced during sulfur deficiencies (Maruyama-Nakashita et al., 2006; Lewandowska et al., 2010). *A. thaliana* has four members (LSU1-4), but homologs can be found in all higher land plants (Sirko et al., 2015; Garcia-Molina et al., 2017). LSU1 was identified as a hub_50_ protein with 80 interactors in AI-1, while 37 interactors were found for LSU2 (Supplementary Table 1) and more than 100 interactors were later identified for LSU3 in the expanded PPIN-1 network (Arabidopsis Interactome Mapping Consortium, 2011; Mukhtar et al., 2011). For LSU4 no interaction data are as yet available, but it is likely that all members of this protein family have hub potential. They have no specific binding domains, but their structure is predicted as a coiled-coil, allowing for flexibility and facilitating protein binding (Sirko et al., 2015).

**Figure 8:**
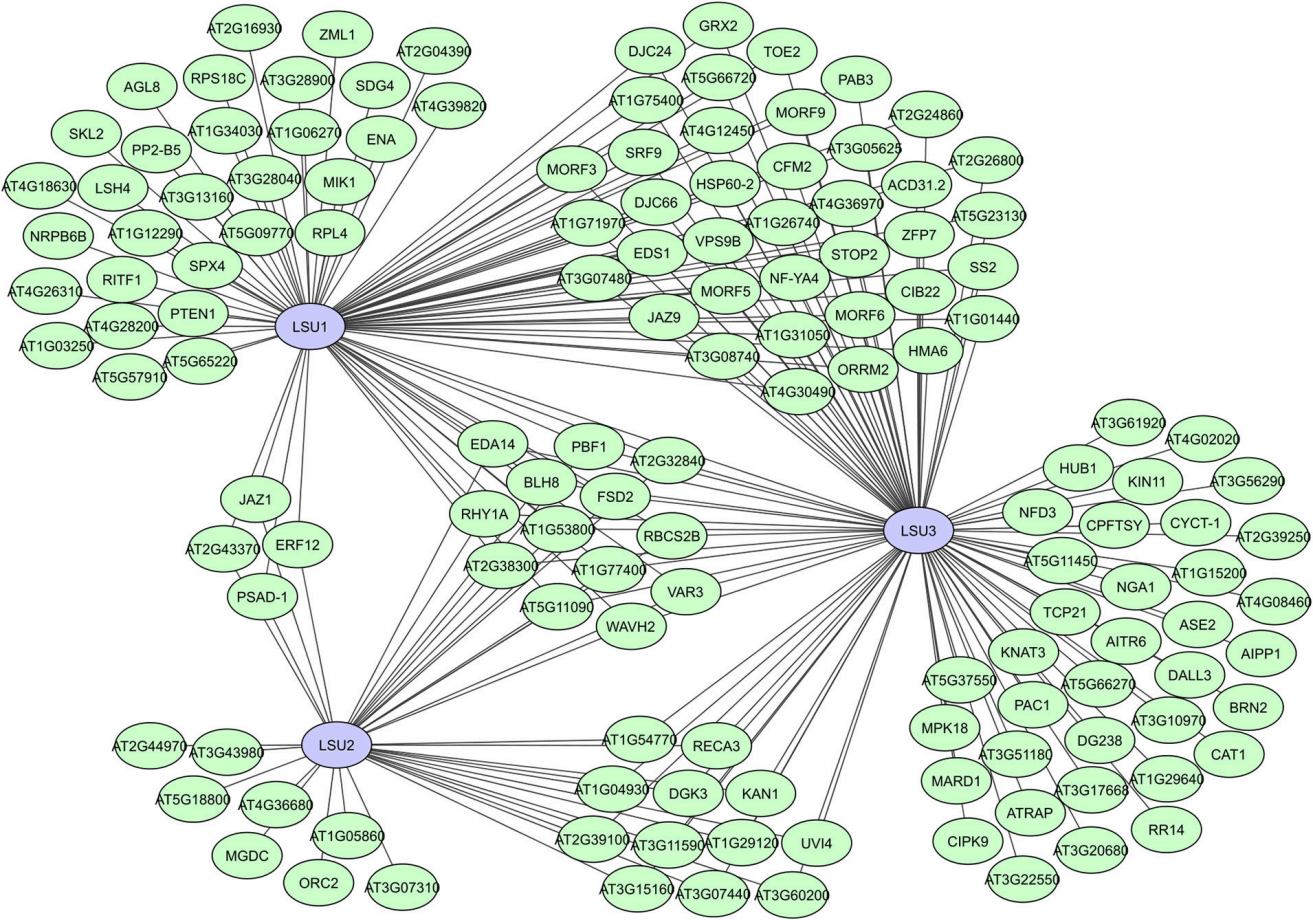
Local interactome for the *A. thaliana* LSU-like proteins LSU1, LSU2, and LSU3. Network based on data from the interactome studies of the Arabidopsis Interactome Mapping Consortium (2011) and Mukhtar et al. (2011).

Interestingly, identified LSU interactors were characterized as functionally very diverse and located in different cellular compartments, suggesting that LSU proteins have complex regulatory functions in various plant processes, including plant immunity and the abiotic stress response (Mukhtar et al., 2011; Sirko et al., 2015; Garcia-Molina et al., 2017). High sequence similarities and existing overlap in interactors between LSU1, LSU2, and LSU3 (Figure 8) suggest partial overlapping functions, while promotor analyses, unique interactors and differences in mutant phenotypes do assume some functional specificity (Sirko et al., 2015). A recent study suggests that LSU proteins can function as network hubs through integrating abiotic and biotic stress responses via interactions with the Fe superoxide dismutase FSD2 (Garcia-Molina et al., 2017). The putative RNA binding protein AtRAP was also recently shown to interacts with LSU2 in chloroplasts and suppress LSU2 under normal conditions (Wang et al., 2017). In general, different modes of action are expected for the LSU proteins due to their high connectivity and seeming involvement in many plant processes, making them highly interesting hubs and targets for further interactomics-based research.

## Conclusions

Plant systems biology driven research is mainly supported by high-throughput -omics analyses with increasing performance, including proteomics and interactomics approaches. In order to represent this burst of data in a comprehensive way, graphical network presentations are used, with the most highly connected node proteins, being of great importance for network structure, stability and functionality. However, authors do not always follow the same definition to characterize these nodes in PPI networks as “hubs.”

The original definition of a hub in biology is that of an outlier in the degree distribution of a scale-free network, like a protein interaction network. Nowadays a hub protein is usually defined as a highly connected central node in a systematic PPI network. Several studies have resulted in a list of network and structural characteristics that are statistically more often attributed to proteins labeled as hubs compared to non-hubs (Table 1). However, not all hub proteins can be defined by one fixed set of such characteristics and depending on whether they are considered as part of large protein networks or as a separate entities, different properties appear to become more important and defining. Defining a protein as a hub is thus a complex matter, as rather the cumulative effect of having (some of) these properties is important to function as a hub in a given PPI network and be labeled as such. Even the intuitive setting of an interaction degree cutoff for hubs is challenging as either fixed or floating cutoffs can be preferred depending on the analyzed PPI networks. Moreover, the originally stated threshold of more than 5 interactions for defining high-degree nodes in general scale-free PPI networks (Vallabhajosyula et al., 2009), appears very low and thus inappropriate for comprehensive PPI networks, while alternatively choosing floating cutoffs also seems rather subjective and depending on the size and connectivity of the network. As such, the setting of a degree cutoff for hub proteins is complex and should be considered with care. There has also been some debate on correlations between degree, essentiality, centrality and pleiotropy, but currently the latter two are generally considered as inherent hub protein characteristics (Yu et al., 2008).

Nevertheless, the term “hub” has recently been used more frequently, and often incorrectly, to loosely label any central signaling network protein of some importance, independent of its connectivity, resulting in some justified confusion between researchers from different fields. However, as there is seemingly a growing emergence of proteins being labeled as hubs, a possible solution could be to make a distinction between signaling hubs (based on their involvement in numerous signaling pathways) and degree hubs (based on their high physical connectivity), potentially further dividing this last group in classes based on the number of interactions, like for example, small (6–10), intermediate (11–50), major (51–100), and super degree hubs (>100 interactions). Additionally, a clear choice should be made to define a PPI network hub as a single highly connected protein and not as a group of several different highly interconnected proteins, as stated by some studies. Here, the terms hub complex or hub module could be used. Nonetheless, new or improved hub classification systems should be considered and validated by network analysis experts, to cope with the boost of novel PPI network data. As hubs have great potential in further unraveling the intricacies of complex biological processes and networks, more clarity and consensus regarding their definition, characteristics and classifications will undoubtedly improve the growing systems biology based research.

In the plant interactome field, early breakthroughs were the first large-scale interolog-based computational *A. thaliana* interactome published by Geisler-Lee et al. (2007) and the high-throughput Y2H-based *A. thaliana* interactomes published by the Arabidopsis Interactome Mapping Consortium (2011) and Mukhtar et al. (2011). Later similar interolog-based studies were performed for other plant species (Tables 2, 3) and more large-scale experimental studies reported, both general and more specific, although only some defined or mentioned hub proteins (Tables 2, 4). In addition, during the last decade major technological advances have been made in the plant interactome field. These include improvements to computational predictions and experimental PPI identifications techniques, as well as improved literature curation of low-throughput experimental studies and increased data from high-quality and high-throughput experimental PPI studies deposited in public PPI databases. An array of databases, tools and resources have been developed to easily retrieve, analyze and visualize plant PPI and other related data (Table 5).

Plant PPI data can thus be obtained from several (complementary) sources, but it is important to realize that the potential of these data in defining hub proteins is strongly dependent on the quality and origin of the data. For instance, networks derived from computational approaches are mostly limited by what is already known in literature (e.g., interolog mapping) and therefore often biased and not accurately representing the biological reality. On the other hand, in networks resulting from experimental approaches, correct hub identification is strongly dependent both on the properties of the applied identification techniques and the experimental scale. Regarding the latter, for example, conclusions drawn from small-scale PPI studies might suffer from a research bias to the protein(s) of interest, resulting in a relatively higher number of identified interaction partners. Hubs are therefore preferably identified in large-scale PPI networks combined with comprehensive PPI networks assembled from curated databases. Still, the degree of proteins identified as hubs can also vary significantly between studies and resulting databases, mainly due to differences in applied techniques, their achieved interaction reliabilities and the risk of focusing on only a subset of the available data. It is also essential to keep in mind that every PPI identification technique has its own strengths and weaknesses. For example, approaches such as AP-MS, used for the identification of plant protein complexes, are very powerful methods but the resulting networks can hardly be used to define general hub proteins as protein degree is affected by preferred investigation and complex size, resulting in a network that is not scale-free. The advantages and disadvantages of different PPI identification techniques and their complementarity have been extensively reviewed (Morsy et al., 2008; Braun et al., 2013; McCormack et al., 2015) and a combination of techniques remains highly recommended for a more accurate PPI validation.

Though at present, various plant proteins have specifically been identified as hub proteins in plant PPI studies, the overlap in identified hubs and their interaction degree is often remarkably small. This becomes especially apparent when comparing hub proteins identified in large-scale computational and experimental PPI studies (Supplementary Tables 1, 2). However, as our knowledge grows and technology improves, it is expected that the gaps between the computationally and experimentally defined hubs will narrow. When looking at all the currently available plant PPI data, already similar (classes of) plant proteins are increasingly being characterized as important plant hub proteins, many of which with a reported role in the plant response to various types of stresses. They represent functional categories of transcription factors, kinases and phosphatases, ubiquitin system associated proteins, (co-)chaperones and redox signaling related proteins (Table 6). Furthermore, a significant number of identified plant hubs are still uncharacterized, making them most interesting targets for future research on (novel) plant stress signaling pathways. As interactomics approaches keep improving, this will undoubtedly lead to a more comprehensive identification of hub proteins and a more efficient system biology driven unraveling of complex biological processes, including those underlying the plant stress response.

## Author contributions

All authors listed have made a substantial, direct and intellectual contribution to the work, and approved it for publication.

### Conflict of interest statement

The authors declare that the research was conducted in the absence of any commercial or financial relationships that could be construed as a potential conflict of interest.
