# Supplementary material for: Hub Protein Controversy: Taking a Closer Look at Plant Stress Response Hubs
Source: Front Plant Sci. 2018 Jun 5;9:694. doi: 10.3389/fpls.2018.00694 (PMC5996676; doi:10.3389/fpls.2018.00694)
Supplement: Supplementary file 1 [file Table_1.docx]

Supplementary Table 1. Hub proteins as pathogen effector targets. The fourteen *A. thaliana* hub_50_ proteins from AI-1 and nine other proteins with a degree higher than 20 in AI-1 which are targeted by at least two pathogens, as shown by Mukthar et al. (2011), together with their pathogen effector target interactions and recurrence in the computationally predicted interactome of Geisler-Lee et al. (2007).

| **Protein** | **Annotation** | **degree** | **Pathogen effector Target*** | **in Geisler-Lee study (Degree)** |
| --- | --- | --- | --- | --- |
| AT5G22290 | ANAC089 – Fructose-sensing quantitative trait locus | 222 | Psy | No |
| AT4G19030 | ATNLM1 – Aquaporin | 181 | Psy, Hpa | No |
| AT4G35580 | CBNAC – Calmodulin-binding NAC protein | 160 | Psy, Hpa | No |
| AT1G22920 | CSN5A – COP9 signalosome 5A | 135 | Psy, Hpa, Gor | Yes (10) |
| AT1G27300 | Unknown transmembrane protein | 112 | Hpa | No |
| AT3G47620 | AtTCP14 – TCP transcription factor | 102 | Psy, Hpa, Gor | No |
| AT3G60600 | VAP27-1 – Vesicle associated protein | 88 | Hpa | No |
| AT4G25200 | AtHSP23.6-MITO – Small heat shock protein | 87 | Hpa | No |
| AT3G49580 | LSU1 – Response to low sulfur protein | 80 | Psy | No |
| AT3G27960 | KLCR2 – Kinesin light chain-related 2 | 74 | Psy, Hpa | Yes (2) |
| AT3G02150 | AtTCP13 – TCP transcription factor | 74 | Psy, Hpa, Gor | No |
| AT3G48150 | APC8 – Anaphase-promoting complex subunit | 67 | Psy, Hpa, Gor | Yes (20) |
| AT3G16310 | Mitotic phosphoprotein N end family protein | 63 | Hpa | No |
| AT4G26450 | Unknown protein | 62 | Hpa | No |
| AT1G69690 | AtTCP15 – Transcription factor | 40 | Psy, Hpa, Gor | No |
| AT4G39050 | MKRP2 – Kinesin motor family protein | 40 | Psy, Hpa | No |
| AT4G17680 | S-ribonuclease binding family protein | 39 | Psy, Hpa, Gor | No |
| AT5G24660 | LSU2 – Response to low sulfur protein | 37 | Psy, Hpa, Gor | No |
| AT3G11590 | Golgin family A protein | 29 | Psy, Hpa | No |
| AT3G07780 | OBERON1 – Nuclear PHD finger protein | 27 | Psy, Hpa | No |
| AT4G02590 | UNE12 – bHLH DNA binding superfamily protein | 24 | Psy, Hpa, Gor | No |
| AT5G51910 | AtTCP19 – TCP transcription factor | 24 | Psy, Hpa | No |
| AT3G17860 | JAZ3 – Jasmonate-ZIM-domein protein 3 | 23 | Psy, Hpa, Gor | No |

*Psy = *P. syringae*; Hpa = *H. arabidopsidis*; Gor = *G. oronti*
