# Supplementary material for: Hub Protein Controversy: Taking a Closer Look at Plant Stress Response Hubs
Source: Front Plant Sci. 2018 Jun 5;9:694. doi: 10.3389/fpls.2018.00694 (PMC5996676; doi:10.3389/fpls.2018.00694)
Supplement: Supplementary file 2 [file Table_2.docx]

Supplementary Table 2. Twenty top hubs from the Geisler-Lee (2007) computational study. Limited overlap between early large-scale interactome studies is clear when comparing the degree of the top hub proteins in the computational Geisler-Lee study with their recurrence in AtORFeome2.0 and the experimentally determined AI-1, PPIN-1 and PPIN-2 networks.

| **Protein** | **Annotation** | **Degree** | **Part of AtORFeome2.0^1^** | **Part of AI-1^1^**  **(Degree)** | **Part of PPIN-1^2^**  **(degree)** | **part of PPIN-2^3^**  **(degree)** |
| --- | --- | --- | --- | --- | --- | --- |
| At4g26840 | ATSUMO1 | 172 | No | No | No | No |
| At1g14400 | ATUBC1 | 119 | No | No | No | No |
| At1g80410 | EMB2753 | 115 | Yes | No | No | No |
| At5g02530 | RNA-binding family protein | 112 | Yes | Yes (3) | No | No |
| At5g13780 | NAA10 | 112 | Yes | Yes (1) | No | No |
| At1g02690 | IMPA6 | 108 | Yes | Yes (40) | Yes (1) | No |
| At4g38630 | ATMCB1 | 108 | Yes | No | No | No |
| At5g26680 | FEN1 | 108 | No | No | No | No |
| At4g25630 | ATFIB2 | 107 | Yes | Yes (3) | No | No |
| At3g48750 | CDC2 | 102 | No | Yes (43) | No | No |
| At3g58560 | ATCCR4A | 101 | Yes | Yes (10) | No | No |
| At5g20850 | ATRAD51 | 100 | No | Yes (11) | No | No |
| At1g04730 | CTF18 | 97 | No | No | No | No |
| At3g22590 | CDC73 | 97 | Yes | No | No | No |
| At2g31970 | ATRAD50 | 94 | Yes | No | No | No |
| At1g29990 | PFD6 | 93 | Yes | Yes (5) | Yes (5) | Yes (3) |
| At3g42660 | Transducin family protein | 92 | No | No | No | No |
| At2g34210 | SPT5-1 | 91 | No | No | Yes (1) | No |
| At3g06720 | IMPA1 | 90 | Yes | Yes (40) | Yes (25) | Yes (12) |
| At2g22290 | ATRABH1D | 89 | No | No | No | No |

^1^The Arabidopsis Interactome Mapping Consortium (2011); ^2^Mukhtar et al. (2011); ^3^Weβling et al. (2014)
